# The antibodies 3D12 and 4D12 recognise distinct epitopes and conformations of HLA-E

**DOI:** 10.3389/fimmu.2024.1329032

**Published:** 2024-03-20

**Authors:** Simon Brackenridge, Nessy John, Klaus Früh, Persephone Borrow, Andrew J. McMichael

**Affiliations:** ^1^ Centre for Immuno-Oncology, Nuffield Department of Medicine, University of Oxford, Oxford, United Kingdom; ^2^ Vaccine & Gene Therapy Institute, Oregon Health & Science University, Beaverton, OR, United States

**Keywords:** HLA-E, MHC-E, antibody, epitope, mapping

## Abstract

The commonly used antibodies 3D12 and 4D12 recognise the human leukocyte antigen E (HLA-E) protein. These antibodies bind distinct epitopes on HLA-E and differ in their ability to bind alleles of the major histocompatibility complex E (MHC-E) proteins of rhesus and cynomolgus macaques. We confirmed that neither antibody cross-reacts with classical HLA alleles, and used hybrids of different MHC-E alleles to map the regions that are critical for their binding. 3D12 recognises a region on the alpha 3 domain, with its specificity for HLA-E resulting from the amino acids present at three key positions (219, 223 and 224) that are unique to HLA-E, while 4D12 binds to the start of the alpha 2 domain, adjacent to the C terminus of the presented peptide. 3D12 staining is increased by incubation of cells at 27°C, and by addition of the canonical signal sequence peptide presented by HLA-E peptide (VL9, VMAPRTLVL). This suggests that 3D12 may bind peptide-free forms of HLA-E, which would be expected to accumulate at the cell surface when cells are incubated at lower temperatures, as well as HLA-E with peptide. Therefore, additional studies are required to determine exactly what forms of HLA-E can be recognised by 3D12. In contrast, while staining with 4D12 was also increased when cells were incubated at 27°C, it was decreased when the VL9 peptide was added. We conclude that 4D12 preferentially binds to peptide-free HLA-E, and, although not suitable for measuring the total cell surface levels of MHC-E, may putatively identify peptide-receptive forms.

## Introduction

In contrast to the highly polymorphic classical major histocompatibility complex (MHC) class Ia molecules, the members of the class Ib family (MHC-E, -F, and -H) have fewer alleles and exhibit significantly less polymorphism (1). For the human MHC-E molecule, also known as human leukocyte antigen-E (HLA-E), there are 342 known functional alleles, encoding 140 distinct proteins (International Immunogenetics [IMGT] HLA Database version 3.55). Despite this apparent diversity, many of these alleles have only been reported once, or possess only synonymous or non-coding changes. Only two forms of the HLA-E protein predominate (HLA-E*01:01 and HLA-E*01:03), and the 97 and 91 alleles (respectively) encoding them appear to be under balancing selection in human populations (2). As a result, the diversity in the HLA-E protein is essentially restricted to a single polymorphism at position 107 (arginine in HLA-E*01:01 and glycine in HLA-E*01:03). This polymorphism lies outside of the peptide binding groove and affects the stability of the HLA-E/β2-microglobulin/peptide complex, resulting in higher cell surface expression of HLA-E*01:03 (3).

HLA-E preferentially presents conserved nonamer peptides derived from the signal sequences of MHC class Ia alleles and HLA-G (4–6). HLA-E in complex with these peptides (which typically vary only at positions 7 and 8: VMAPRT[V/L][V/I/L/F]L, VL9) is recognised by the CD94/NKG2 receptors expressed by natural killer(NK) cells and a subset of CD8^+^ T cells (7–9). In addition to presenting this self-peptide, however, there has been a growing realisation that HLA-E can also present peptides from a variety of bacterial and viral pathogens (10). In most infections, T cell responses to HLA-E-bound peptides are rare compared to those recognising peptides presented by classical MHC Ia molecules, but exceptions are known. For example, the CD8^+^ T cells induced by a rhesus cytomegalovirus (CMV) strain 68-1-vectored SIV vaccine that enable ~55% of vaccinated rhesus macaques to clear infection following challenge with SIV_mac239_ (11, 12) are restricted by Mamu-E, the Rhesus macaque orthologue of HLA-E, and MHC class II rather than MHC class Ia (13). The protection conferred by this vaccine depends on these Mamu-E-restricted CD8^+^ T cells (14), and not the MHC class II-restricted CD8^+^ T cells (15). This has raised the prospect of a HIV-1 vaccine that mediates protection via HLA-E-restricted CD8^+^ T cells, and there is now great interest in exploiting HLA-E for both vaccine and immunotherapy strategies for both infectious diseases and tumours (16–20).

With this increased focus on HLA-E, it is important that the available HLA-E antibodies are fully characterised. We (21), and others (22), have previously shown that some of the commercially available HLA-E antibodies are not truly specific. However, the most commonly used HLA-E antibody, 3D12, which was isolated from HLA-B27 transgenic mice immunised with recombinant HLA-E (9), has minimal cross-reactivity with classical MHC class I proteins (21). The laboratory that isolated 3D12 later isolated a second antibody against HLA-E, 4D12 (23). Although 4D12 was reported not to bind to a panel of 60 HLA Ia-diverse cell lines, we are not aware of any systematic analysis of the cross-reactivity of this antibody with conventional MHC class I alleles. In terms of MHC-E reactivity, however, 4D12 differs from 3D12 by staining cells expressing either the rhesus macaque allele Mamu-E*02:04 (13), or the cynomolgus macaque allele Mafa-E*02:01:02 (24). Although only a small number of Mamu-E and Mafa-E alleles have been sequenced (33 and 27, respectively, IMGT MHC Database version 3.12.0.0), both appear to be more polymorphic than HLA-E, which may have implications for 4D12 binding.

In addition to understanding the repertoire of MHC-E alleles that these two antibodies bind, knowledge of the locations of their epitopes will allow determination of whether they are suitable for use in blocking engagement with particular receptors, or whether biotinylation of exposed lysine residues in or near their binding sites could prevent antibody binding. Knowledge of the epitopes may also be critical to understand what external factors could alter interaction of the antibodies. It has recently been shown that the binding of the pan-MHC class I antibody W6/32 can, in certain situations, be influenced by the lipid composition of the plasma membrane (25). Membrane compositions can differ significantly between cell types and activation states (26), so knowing precisely where an antibody binds on a cell surface protein may be essential to the correct interpretation of staining patterns.

3D12 and 4D12 recognise different regions of HLA-E (23), but the epitopes of these antibodies have never been properly mapped. An early attempt to map the 3D12 epitope using peptides to block binding suggested two regions on the HLA-E extra-cellular domain (27). As both of the sequences (on the floor of the peptide binding groove, and at the start of the alpha 2 domain) are conserved in Mamu-E*02:04, which is not recognised by 3D12, neither is likely to comprise the epitope. A later attempt to map the 3D12 epitope using overlapping peptides spanning the HLA-E protein was also unsuccessful, and it was concluded that the 3D12 epitope has some dependence on conformation (28).

To map the epitope of 3D12, we exploited the fact that this antibody binds HLA-E but not Mamu-E. Staining of cells expressing hybrids of HLA-E*01:03 and Mamu-E*02:04 identified a short sequence unique to HLA-E on the alpha 3 domain that was critical for 3D12 to bind. For 4D12, staining of cells expressing hybrids of Mamu-E*02:04 and a second Mamu-E allele (Mamu-E*02:16) that is not recognised by 4D12, allowed us to map the 4D12 epitope to the start of the MHC-E alpha 2 helix, adjacent to the C terminus of the presented peptide. We also confirmed that 4D12, like 3D12, shows little cross-reaction with a panel of classical HLA-ABC alleles. Finally, we observed that staining with these two antibodies was differentially affected when cells over-expressing HLA-E were incubated with exogenous peptides. While staining with 3D12 was increased by the presence of peptide, that of 4D12 was decreased. These results are consistent with 4D12 preferentially binding to peptide-free HLA-E and suggest that staining with 4D12 may not reflect the levels of peptide-presenting MHC-E on the cell surface.

## Methods

### Plasmids & cloning

Plasmids expressing the single chain trimers (SCT) of Mamu-E*02:04 and HLA-E*01:03 with the VL9 peptide (VMAPRTLLL) were as previously described (13 and 29, respectively). Additional single chain trimers of HLA-E*01:01 and Mamu-E*02:16 were created using an identical cloning strategy. Hybrids of the SCT constructs were made using restriction enzymes (PpuMI, Bsu36I, and SbfI), as described in the Results section. Mutations of specific codons in HLA-E and Mamu-E were introduced by overlap-extension PCR (30) using the primers and templates indicated in **Supplementary Tables 1-3**, and the resulting mutated sequences inserted into the relevant expression plasmids using the PpuMI and SbfI restriction sites. The plasmids expressing the single chain dimers of β2-microglobulin linked to HLA-E*01:03, HLA-A*02:01, HLA-A*11:01, HLA-B*40:06, HLA-C*17:01 and HLA-C*18:02 were created by inserting the mature coding sequences into a plasmid expressing a single chain dimer of HLA-E*01:01 (13), using BamHI restriction sites engineered at the start and end of the HLA coding sequences. The β2-microglobulin/Enhanced Green Fluorescent Protein (EGFP) expression plasmid was created by inserting a sequence encoding β2-microglobulin followed by the picornavirus 2A “slip” sequence (GSGATNFSLLKQAGDVEENPGP) in to pEGFP-N1 (Clontech) using the HindIII and AgeI restriction sites. All plasmids were prepared using QIAprep mini spin columns (QIAGEN) and verified by Sanger sequencing using an ABI3770.

### Cell culture, transfection & staining

HEK 293T cells were maintained between 10% and 90% confluency at 37°C/5% CO_2_ in high glucose Dulbecco’s Modified Eagle’s Medium (DMEM, Life Technologies) with L-glutamine (Life Technologies) supplemented with 10% foetal bovine serum (FBS, Sigma), and penicillin/streptomycin (50 units/ml and 50 µg/ml, respectively; Life Technologies). Transfections of 293T cells were carried out in 6 well plates using GeneJuice (Merck) with 1 μg of plasmid DNA per well, as per the manufacturer’s instructions. K562 cells over-expressing HLA-E*01:03 (31) (K562E, a kind gift of Thorbald van Hall, Leiden University Medical Centre, Netherlands) were maintained 37°C/5% CO_2_ between 3x10^5^ and 2x10^6^ cells per ml in Iscove’s Modified Dulbecco’s Medium (IMDM, Life Technologies), supplemented with L-glutamine (Life Technologies), 8% FBS and penicillin/streptomycin (as above).

### Staining & flow cytometry

293T cells were stained 24 hours post transfection with 3D12 (BioLegend), 4D12 (MBL), or 2M2 (BioLegend) at 10 ng/μl (or the concentration indicated in the figure) in 100µl of Dulbecco’s Phosphate Buffed Saline (DPBS, Sigma) at 4°C for 15 minutes, washed twice with DPBS, stained with secondary antibody (allophycocyanin-crosslinked Goat-Anti-Mouse (H+L) F(ab’)2 fragment, Life Technologies) in 100µl of DPBS for 15 minutes at 4°C, washed as before, and fixed in 100µl of Cytofix (BD Biosciences). For the cross-blocking experiments, cells were pre-incubated for 20 minutes at 4°C with the stated concentrations of 3D12, 4D12 or an irrelevant antibody (MA2.1), then stained with 3D12-APC (BioLegend) diluted 1:1000 in 100µl of DPBS at 4°C for 15 minutes. Cells were then washed twice with DPBS and fixed as before. Stained cells were acquired using a CyAn ADP Analyser (Beckman Coulter) or an Attune NXT (Life Technologies) and analysed using the gating strategies shown in **Supplementary Figure 1A** (293T cells) and **1B** (K562E), using FlowJo 10 (BD).

### β2-microglobulin CRISPR

The β2-microglobulin-specific guide RNA 5’-CGCGAGCACAGCTAAGGCCA-3’ (32), which targets the reverse strand of the first exon, was inserted into pspgRNA (33, a gift from Charles Gersbach [Addgene plasmid # 47108; http://n2t.net/addgene:47108; RRID: Addgene_47108]), with an additional guanosine included at the 5’ end to ensure efficient transcription. 293T cells were co-transfected with equal amounts of this plasmid and pCas9_GFP (34, a gift from Kiran Musunuru [Addgene plasmid # 44719; http://n2t.net/addgene:44719; RRID: Addgene_44719]). EGFP-positive cells were sorted 48 hours post transfection on a MoFlo cell sorter (Beckman Coulter), expanded after limiting diluting in 96 well plates, and 8 clones that were MHC class I negative by surface staining with W6/32 grown out. We were unable to confirm the exact nature of the genetic lesion introduced into the genomic DNA of these clone as PCR primers flanking exon 1 of the β2-microglobulin gene (5’-AGAATGAGCGCCCGGTGTCC-3’ and 5’-CCCGCCGAAAGGGGCAAGTA-3’) failed to amplify any product with genomic DNA from any of the clones, suggesting significant disruption to the start of the gene. One clone which grew and transfected well was selected for use (**Supplementary Figure 2A**). Transfection of these cells with a plasmid expressing β2-microglobulin was sufficient to restore surface MHC class I expression (**Supplementary Figure 2B**), confirming that the loss of W6/32 staining was the result of disruption of the β2-microglobulin gene.

### Characterisation of antibody cross-reactivity

LABScreen Single Antigen Class I Combi beads (One Lambda), each coated with a single HLA allotype, were incubated with 3D12, 4D12 or W6/32 (BioLegend) for 30 minutes at room temperature, washed 3 times with PBS, incubated at room temperature for 30 minutes with goat anti-mouse IgG-R-PE (Invitrogen), washed 3 times as before, and analysed on a LABScan 100 flow analyser. Binding of each antibody was corrected by subtracting the fluorescence of the negative control beads, and the binding of 3D12 and 4D12 was normalised relative to the binding of W6/32, to control for variation in the amount of correctly folded protein for each HLA allele.

### Peptide pulsing

Synthetic VL9 peptide (VMPARTLVL) was generated by Fmoc (9-fluorenylmethoxy carbonyl) chemistry to a purity of 85% (Genscript, Hong Kong). Lyophilized peptide was reconstitution to a final concentration of 200mM in dimethyl sulfoxide (DMSO), aliquoted, and stored at -80°C. Following overnight incubation at either 37°C or 27°C (to increase surface expression of HLA-E), 6x10^5^ K562E cells in 300 μl of supplemented IMDM were mixed with an equal volume of supplemented IMDM containing VL9 peptide at a concentration of 0 (DMSO only), 20 or 200 μM and incubation at 37°C or 27°C continued. After 4 hours, 5x10^5^ cells were stained with 3D12 and 4D12 as above, except DPBS supplemented with 0.2% bovine serum albumin (BSA, Sigma) was used for all incubations and washes, and cells were gated as shown in **Supplementary Figure 1B**.

### Data analysis

Data were analysed using Prism 10.0.2 (GraphPad). Normal distribution of the data was confirmed using the Kolmogorov-Smirnov and Shapiro-Wilk tests (depending on sample size). Statistical significance was determined using the tests indicated in the figure legends.

## Results

### MHC-E allele reactivity of the commercially available HLA-E antibodies

**Figure 1:**
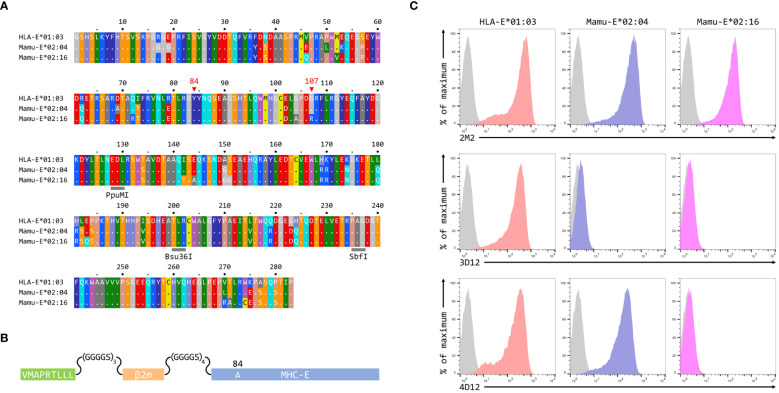
Reactivity of 3D12 and 4D12 with the alleles used in this study. **(A)** Sequences of the extracellular domains (ECD) of HLA-E*01:03, Mamu-E*02:04, and Mamu-E*02:16. Sequence identity is indicated by dots and amino acids are coloured according to the standard scheme used by RasMol. Positions 107 (the predominant polymorphism in HLA-E), and the conserved tyrosine at position 84 (mutated to alanine in the SCT to open the end of the peptide binding groove) are indicated. The approximate locations of the three restriction sites used to create the hybrids of the different alleles are also highlighted. **(B)** Schematic of the SCT constructs, which encode the peptide VMAPRTLLL, a flexible glycine-serine linker (GGGGSx3), the mature β2-microglobulin coding sequence, a second flexible linker (GGGGSx4), and the mature coding sequences of HLA-E*01:03, Mamu-E*02:04, or Mamu-E*02:16. The constructs also include the coding sequence of EGFP at the C terminus (not shown) to allow gating of transfected cells. **(C)** Representative staining (of at least five independent replicates) with 2M2 (a β2-microglobulin-specific antibody, top row), 3D12 (middle row), or 4D12 (bottom row), of β2-microglobulin-deficient 293T cells transfected with plasmids expressing the HLA-E*01:03 SCT (first column), the Mamu-E*02:04 SCT (central column), or the Mamu-E*02:16 SCT (final column). Grey histograms show staining of mock transfected cells.

**Figure 2:**
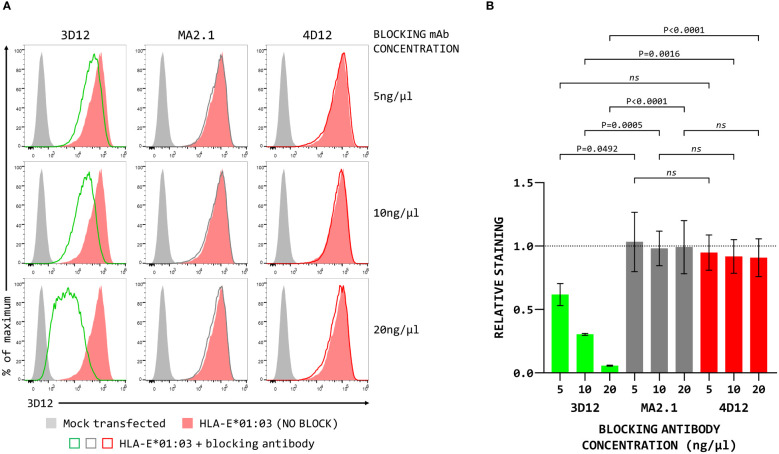
Binding of 4D12 does not block binding of 3D12. **(A)** Representative staining (of three independent replicates) with APC-conjugated 3D12 with APC of β2-microglobulin-deficient 293T cells transfected with plasmids expressing the HLA-E SCT following pre-incubation with unconjugated 3D12 (left column), an isotype control antibody (MA2.1, middle column) or 4D12 (right column) at 5ng/μl (top row), 10ng/μl (middle row), or 20ng/μl (bottom row). Mock transfected cells are shown as filled grey histograms, cells without antibody pre-incubation as filled red histograms, and cells stained with 3D12-APC as the coloured lines. **(B)** Quantification of the three independent replicates with staining normalised to that of the cells without anti-body pre-incubation. Statistical analysis was by one way ANOVA with Tukey’s correction for multiple comparisons. P values are indicated; ns, not significant.

To map the binding sites of the two antibodies on HLA-E we employed three different alleles of MHC-E: HLA-E*01:03, Mamu-E*02:04, and Mamu-E*02:16. The sequences of the extracellular domains of these proteins are shown in **Figure 1A**. As HLA-E is normally expressed at very low levels by most cells, we expressed these as single chain trimers (SCT) of the MHC protein, β2-microglobulin (β2m) and the VL9 peptide, linked by flexible glycine-serine linkers (**Figure 1B**). We have previously observed poor staining with W6/32 of cells expressing SCT of a number of different MHC class I alleles (**Supplementary Figure 3**), perhaps because the linker between the peptide and the N terminus of β2m hinders access of W6/32 to its epitope, which is known to involve position 3 of β2m (35). Therefore, an antibody against β2m (2M2) was used to confirm surface expression of all three MHC-E SCT (**Figure 1C**, top row). 3D12 and 4D12 (**Figure 1C**, middle and bottom rows, respectively) both stained cells expressing the HLA-E*01:03 SCT, while 4D12 also stained cells expressing the Mamu-E*02:04 SCT. Neither antibody stained cells expressing the Mamu-E*02:16 SCT. We also confirmed that these antibodies do indeed recognise distinct epitopes, as previously reported (23). Pre-incubation of cells transfected with the HLA-E*01:03 SCT with a either an isotype control antibody (MA2.1; 36) or 4D12 at three different concentrations had no effect on subsequent staining with 3D12 (**Figure 2A**, middle and right-hand columns). In contrast, pre-incubation of cells with the same concentrations of 3D12 had a significant effect on subsequent staining with 3D12 (**Figure 2A**, left hand column; **Figure 2B**).

### Mapping the primary contacts of 3D12

**Figure 3:**
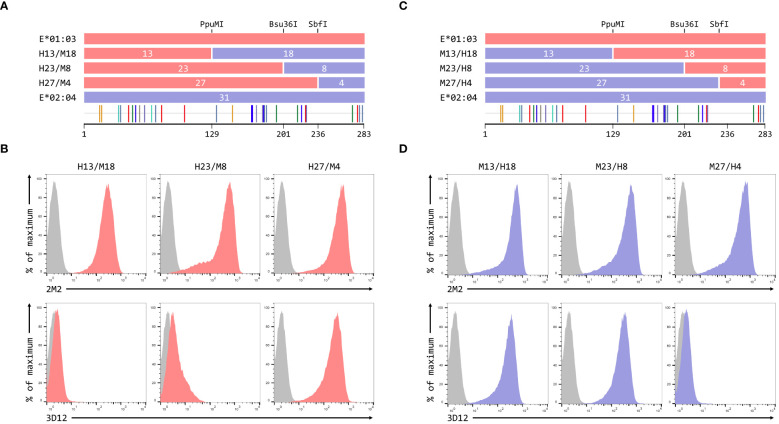
Localisation of residues critical for the binding of 3D12. **(A, C)** Schematics of the ECD of the HLA-E*01:03/Mamu-E*02:04 **(A)** or Mamu-E*02:04/HLA-E*01:03 **(C)** hybrids a used to map the 3D12 epitope, which were created using the conserved PpuMI, Bsu36I or SbfI restriction sites. The highlighter plots at the bottom indicate the locations of the 31 positions that differ between the extracellular domains of these two alleles, and the number of these on either side of each restriction site (13 and 18, 23 and 8, or 27 and 4) are indicated. **(B, D)** Representative staining (of at least five independent replicates) with 2M2 (top row), or 3D12 (bottom row), of β2-microglobulin-deficient 293T cells transfected with plasmids expressing the HLA-E*01:03/Mamu-E*02:04 **(B)** or Mamu-E*02:04/HLA-E*01:03 **(D)** hybrid SCT. Grey histograms show staining of mock transfected cells.

To map the residues critical for 3D12 to bind to HLA-E, we created a series of reciprocal hybrids of the HLA-E*01:03 and Mamu-E*02:04 SCT (**Figures 3A, C**), using three unique restrictions sites shared by both alleles (PpuMI, Bsu36I, and SbfI). The extracellular domains of these two alleles differ at 31 different positions, with the hybrids having 13, 23 and 27 of these polymorphisms swapped. We denoted these hybrids as H13/M18 and M13/H18, H23/M8 and M23/H8, or H27/M4 and M27/H4, depending on whether the human or macaque sequence was first. Staining with 2M2 confirmed that all hybrids expressed well at the cell surface (**Figures 3B, D**, top row). For the human/macaque hybrids, only cells expressing H27/M4 were stained well by 3D12, with no staining observed for cells expressing H13/M18 and only very weak staining of cells expressing H23/M8 (**Figure 3B**, bottom row). Conversely, good staining was seen with cells expressing two of the macaque/human hybrids: M13/H18 and M23/H8 (**Figure 3D**, bottom row).

**Figure 4:**
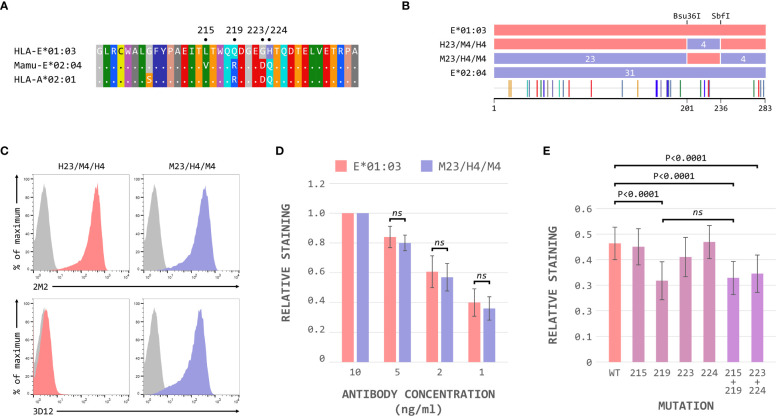
The polymorphisms at positions 219, 223 and 224 are critical for 3D12 binding to HLA-E. **(A)** Comparison of the sequence between the Bsu36I and SbfI restriction sites (positions 200 to 236) of HLA-E*01:03, Mamu-E*02:04, and HLA-A*02:01. Sequence identity is indicated by dots and amino acids are coloured according to the standard scheme used by RasMol. **(B)** Schematic of the ECD of the HLA-E*01:03/Mamu-E*02:04 (H23/M4/H4) and Mamu-E*02:04/HLA-E*01:03 (M23/H4/M4) hybrids, which have the sequence between the Bsu36I and SbfI restriction sites swapped. The highlighter plot at the bottom indicates the locations of the 31 positions that differ between the extracellular domains of these two hybrids. **(C)** Representative staining (of at least 5 replicates) with 2M2 (top row), or 3D12 (bottom row), of β2-microglobulin-deficient 293T cells transfected with plasmids expressing the H23/M4/H4 (left column) or M23/H4/M4 (right column) hybrid SCT. Grey histograms show staining of mock transfected cells. **(D)** Staining using decreasing amounts (10ng/μl, 5ng/μl, 2ng/μl and 1ng/μl) of 3D12 of β2-microglobulin-deficient 293T cells transfected with plasmids expressing the HLA-E*01:03 (left) or M23/H4/M4 (right) hybrid SCT. The graph shows the normalised 3D12 staining (mean ± SD) for four independent replicates, with staining using the highest amount of antibody set to 1 for each SCT. Statistical significance was assessed by unpaired two-tailed Student’s t tests. **(E)** Staining of cells expressing the HLA-E SCT mutated at positions L215V, Q219R, G223D, H224Q, L215V and Q219R, or G223D and H224Q. The graph shows mean ± SD 3D12 staining (with 10ng/μl antibody) corrected for surface expression (2M2 staining, 10 ng/μl), for seven independent replicates. Statistical analysis was performed using a repeated measures ANOVA with Dunnett’s correction for multiple comparisons. P values are indicated; ns, not significant.

These results suggested that the region between the Bsu36I and SbfI restrictions sites, containing the polymorphisms at positions 215, 219, 223 and 224 on the alpha 3 domain, is critical for the binding of 3D12 to HLA-E. The sequence of Mamu-E*02:04 across this region more closely resembles that of HLA-A*02 (and the majority of classical MHC class I alleles) than HLA-E (**Figure 4A**), differing only in the presence of valine rather than leucine at position 215. In contrast, HLA-E only matches classical MHC class I alleles at position 215, with the combination of amino acids at positions 219, 223 and 224 being unique to HLA-E.

Hybrids of HLA-E*01:03 and Mamu-E*02:04 with these four polymorphisms swapped (H23/M4/H4 and M23/H4/M4, **Figure 4B**) confirmed that this region was indeed critical for the binding of 3D12: only cells expressing the M23/H4/M4 hybrid stained with 3D12 (**Figure 4C**). Comparable reductions in the relative staining of cells expressing the M23/H4/M4 hybrid and HLA-E*01:03 when decreasing concentrations of 3D12 were used (**Figure 4D**), confirmed that the hybrid contained the full epitope. When each of the four polymorphisms were mutated individually, however, a reduction in 3D12 staining was only observed with the Q219R change (**Figure 4E**). Combination of the G223D and H224Q mutations also resulted in a reduction in 3D12 staining, while the combination of L215V and Q219R was no more detrimental that the Q219R mutation alone. Therefore, we conclude that the polymorphisms at positions 219, 223 and 224 are the reason for the lack of staining of Mamu-E*02:04 with 3D12.

In summary, our results locate the epitope of 3D12 on the alpha 3 domain of HLA-E, in the vicinity of positions 219–224. Further work is required to identify the exact residues in this region that are directly contacted by the antibody or are important for maintaining the correct conformation of the epitope.

### Mapping the primary contacts of 4D12

**Figure 5:**
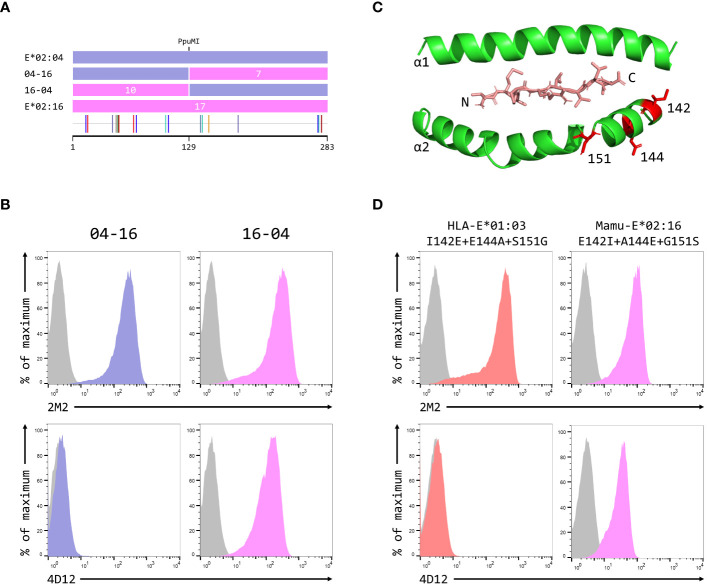
Identification of residues critical for the binding of 4D12. **(A)** Schematic of the ECD of the hybrids of Mamu-E*02:04 and Mamu-E*02:16 used to map the region bound by 4D12, which were created using the PpuMI restriction site. The highlighter plot at the bottom indicates the locations of the 17 positions that differ between the ECD of these two alleles, 10 of which lie upstream of the hybrid junction. **(B)** Representative staining (of at least five independent replicates) with 2M2 (top row), or 4D12 (bottom row), of β2-microglobulin-deficient 293T cells transfected with plasmids expressing the 04-16 (left column) or 16-04 (right column) hybrid SCT. **(C)** Location in HLA-E of the three polymorphisms (positions 142, 144 and 151) most likely to encompass the location of the 4D12 epitope (PDB: 1MHE). The VL9 peptide is shown in stick form, while the alpha 1 and alpha 2 domains of HLA-E are shown as ribbons, with the side chains of the relevant polymorphic positions also included. **(D)** Representative staining (of at least five independent replicates) with 2M2 (top row), or 4D12 (bottom row), of β2-microglobulin-deficient 293T cells transfected with plasmids expressing the HLA-E*01:03 SCT with positions 142, 144 and 151 mutated to the amino acids present in Mamu-E*02:16 (left column), the Mamu-E*02:16 SCT with positions 142, 144 and 151 mutated to the amino acids present in HLA-E*01:103 (right column). In panels **(B, D)**, grey histograms show staining of mock transfected cells.

To map the epitope of 4D12, we first created reciprocal hybrids of the Mamu-E*02:04 and Mamu-E*02:16 SCT, using the PpuMI restriction site within codons 128–131 of the extracellular domains (**Figure 5A**). These two alleles of Mamu-E differ at 17 positions in their extra-cellular domains, with 10 of the differences being present upstream of the hybrid junction. Staining with 2M2 confirmed that both hybrid SCT expressed well at the cell surface (**Figure 5B**, top row), but only cells expressing the hybrid with Mamu-E*02:16 sequence first (designated 16-04) stained well with 4D12 (**Figure 5B**, bottom row). Thus, the critical amino acids that allow 4D12 to bind to Mamu-E*02:04 must include one or more of the 7 polymorphisms (142, 144, 151,183, 270, 271 and 274) downstream of the hybrid junction. Of these, the region encompassing the final three polymorphisms (positions 270, 271 and 274) is more conserved between Mamu-E*02:04 and HLA-A*02:01 (**Supplementary Figure 4A**) which is not bound by 4D12 (**Supplementary Figure 4B**) that it is between Mamu-E*02:04 and HLA-E*01:03. Similarly, a lack of conservation between Mamu-E*02:04 and HLA-E in the sequences flanking the polymorphism at position 183, which lies between the alpha 2 and alpha 3 domains, suggested that this region was also unlikely to be relevant for 4D12 binding (**Supplementary Figure 4C**).

Therefore, we focused our attention on the positions 142, 144 and 151. The first two of these lie at the N-terminal end of the alpha 2 helix, adjacent to the C terminus of the presented peptide, while 151 lies between the alpha 2-1 and 2-2 helices (**Figure 5C**). Combined mutation of all three of these positions in the HLA-E*01:03 SCT (to the residues present in Mamu-E*02:16) did not affect cell surface expression of the HLA-E*01:03 SCT (**Figure 5D**, left-hand column, top row), but completely abolished staining with 4D12 (**Figure 5D**, left-hand column, bottom row). In contrast, although introduction of the opposite mutations into the Mamu-E*02:16 SCT reduced surface expression of the Mamu-E*02:16 SCT (**Figure 5D**, right-hand column, top row), cells expressing this SCT were stained by 4D12 (**Figure 5D**, right-hand column, top row). These results identify the region encompassing positions 142, 144 and 151 as critical for the binding of 4D12.

**Figure 6:**
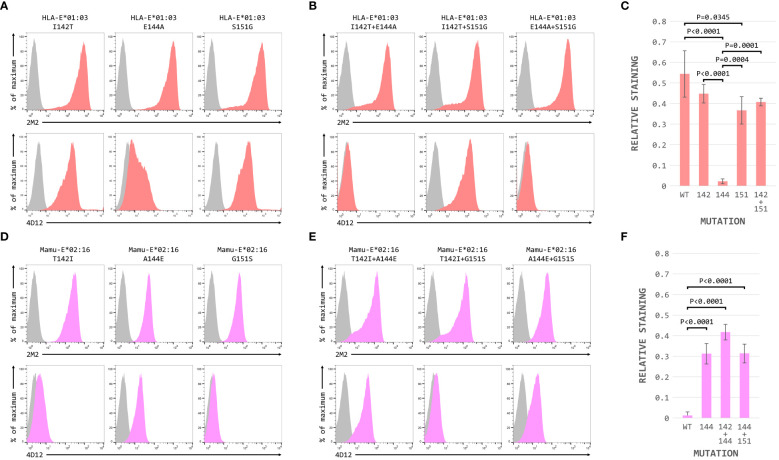
Positions 142, 144 and 151 are important for the binding of 4D12. Representative staining (of at least five independent replicates) with 2M2 (top row), or 4D12 (bottom row), of β2-microglobulin-deficient 293T cells transfected with plasmids expressing **(A)** the HLA-E*01:03 SCT with positions 142 (left column), 144 (central column) or 151 (last column) individually mutated to the amino acids present in Mamu-E*02:16; **(B)** the Mamu-E*02:04 SCT with positions 142 (left column), 144 (central column) or 151 (last column) individually mutated to the amino acids present in HLA-E-E*01:03; **(D)** the HLA-E*01:03 SCT with positions 142 and 144 (left column), 142 and 151 (central column) or 144 and 151 (last column) mutated to the amino acids present in Mamu-E*02:16; or **(E)** plasmids expressing the Mamu-E*02:04 SCT with positions 142and 144 (left column), 142 and 151 (central column) or 144 and 151 (last column) mutated to the amino acids present in HLA-E-E*01:03. In all panels, grey histograms show staining of mock transfected cells. Panels **(C, F)** show quantification of selected transfections for 3 independent replicates (mean ± SD, corrected for surface expression using the 2M2 staining). Statistical analysis was performed using a one-way ANOVA with Tukey’s correction for multiple comparisons.

When these three positions were mutated individually in the HLA-E*01:03 SCT, only the E144A change had a marked effect on 4D12 staining (**Figure 6A**, bottom row; **Figure 6C**). 4D12 staining was only modestly affected by combination of the I142T and S151G mutations (**Figure 6B**, bottom row, column 2; **Figure 6C**). Despite this, positions 142 and 151 are important for the binding of 4D12: combination of E144A with either I141T or S151G (**Figure 6B**, bottom row, columns 1 and 3, respectively) resulted in the complete loss of 4D12 staining. Interpretation of the effects of the opposite mutations introduced into the Mamu-E*02:16 SCT was complicated by the fact that some of the mutations again affected both cell surface expression of the SCT as well as 4D12 binding (**Figures 6D, E**). However, the A144E mutation was the only single mutant that allowed 4D12 binding (**Figure 6D**, bottom row, middle column; **Figure 6F**), and the combination of T142I with G151S was not sufficient to allow 4D12 to bind (**Figure 6E**, bottom row, middle column).

The dramatic effect on 4D12 binding mediated by the various single and double mutations, in contrast to the more subtle effects of individual mutations on the binding of 3D12 (**Figure 4E**), is consistent with these positions playing a direct role in the binding of 4D12 to what is known to be a conformationally-independent epitope. Although the contributions of the other (conserved) residues in this region remain to be determined, we conclude that the glutamate at position 144 is critical for 4D12 binding. In addition, the isoleucine at position 142 and the serine at position 151 also contribute to the stability of the bound antibody, although these two positions are insufficient alone or in combination to allow stable 4D12 binding.

### Cross-reactivity of 4D12 and 3D12 with classical MHC class I alleles

**Figure 7:**
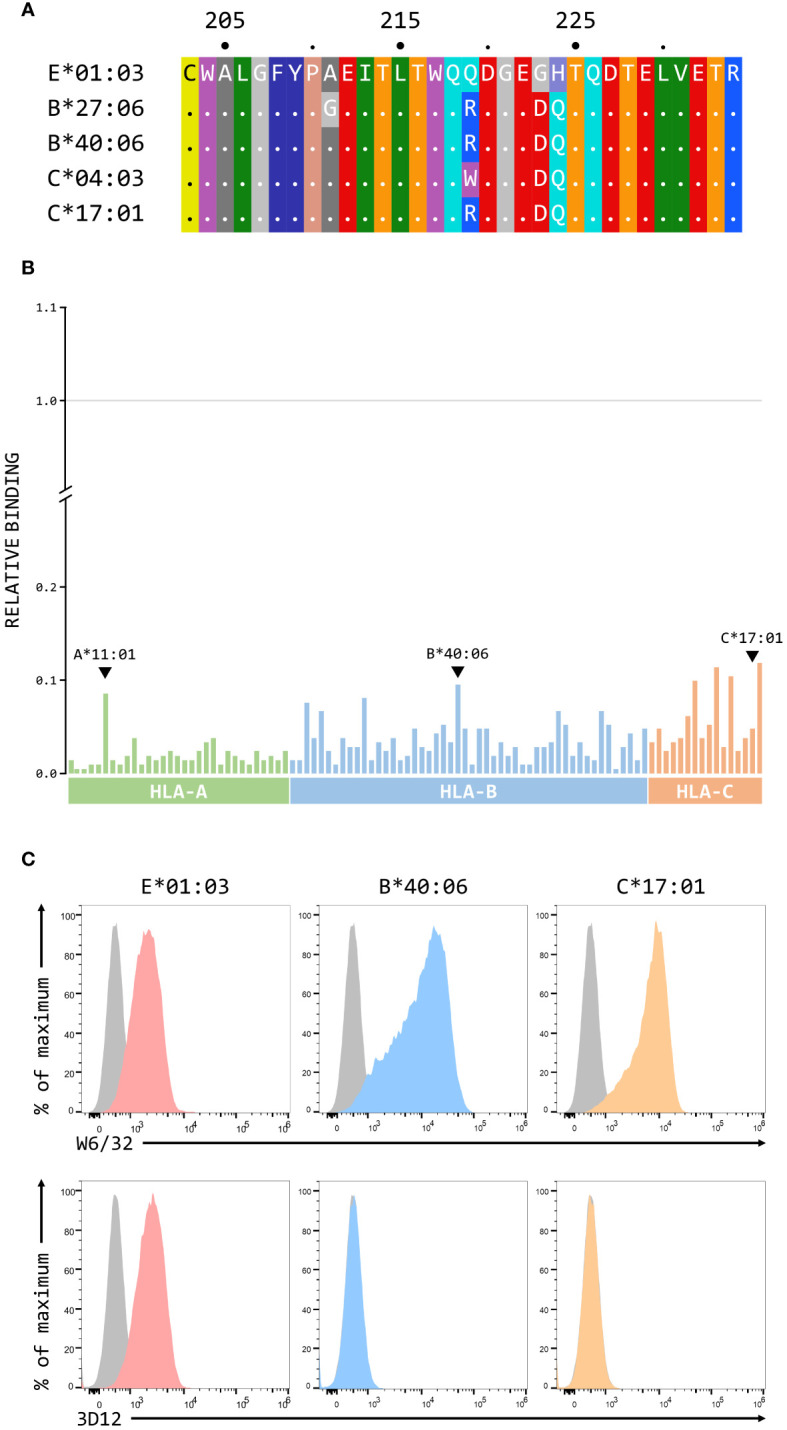
3D12 does not cross-react with classical MHC class I proteins. **(A)** Comparison of the sequences of the region encompassing the 3D12 epitope for HLA-E*01:03 and the 4 classical HLA alleles reported to cross-react with 3D12. Sequence identity is indicated by dots and amino acids are coloured according to the standard scheme used by RasMol. **(B)** Binding of 3D12 to beads coated with recombinant HLA-A (green), B (blue) and C (red) protein, normalised relative to the binding of the pan-HLA antibody W6/32 (set to 1). The allotypes tested were: HLA-A*01:01, 02:01, 02:03, 02:06, 03:01, 11:01, 11:02, 23:01, 24:02, 24:03, 25:01, 26:01, 29:01, 29:02, 30:01, 30:02, 31:01, 32:01, 33:01, 33:03, 34:01, 34:02, 36:01, 43:01, 66:01, 66:02, 68:01, 68:02, 69:01, 74:01, 80:01; HLA-B*07:02, 08:01, 13:01, 13:02, 14:01 14:02, 15:01, 15:02, 15:03, 15:10, 15:11, 15:12, 15:13, 15:16, 18:01, 27:05, 27:08, 35:01, 37:01, 38:01, 39:01, 40:01, 40:02, 40:06, 41:01, 42:01, 44:02, 44:03, 45:01, 46:01, 47:01, 48:01, 49:01, 50:01, 51:01, 51:02, 52:01, 53:01, 54:01, 55:01, 56:01, 57:01, 57:03, 58:01, 59:01, 67:01, 73:01, 78:01, 81:01, 82:01; HLA-C*01:02, 02:02, 03:02, 03:03, 03:04, 04:01, 05:01, 06:02, 07:02, 08:01, 12:03, 14:02, 15:02, 16:01, 17:01, 18:02. **(C)** Representative staining (of at least five independent replicates) with W6/32 (top row), or 3D12 (bottom row), of β2-microglobulin-deficient 293T cells transfected with plasmids expressing single chain dimers (SCD) HLA-E*01:03 (left), HLA-B*40:06 (middle), or HLA-C*17:01 (right). Grey histograms show staining of mock transfected cells.

**Figure 8:**
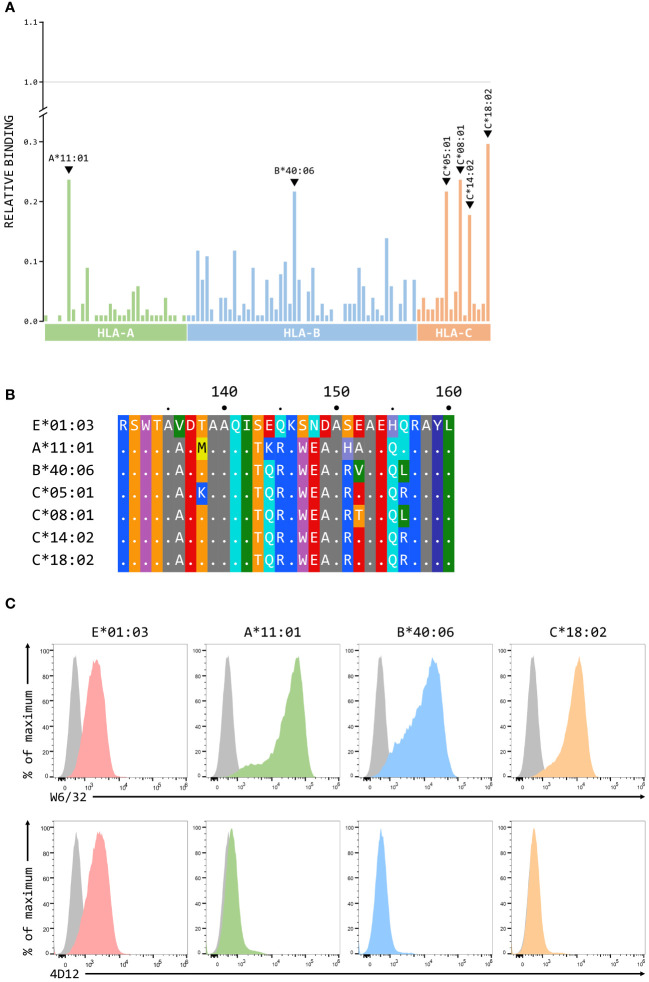
4D12 does not cross-react with classical MHC class I proteins. **(A)** Binding of 3D12 to beads coated with recombinant HLA-A (green), B (blue) and C (red) protein, normalised relative to the binding of the pan-HLA antibody W6/32 (set to 1). The allotypes tested were as **Figure 7**. **(B)** Comparison of the sequences of the region encompassing the 4D12 epitope for HLA-E*01:03 and the 6 classical HLA alleles exhibiting the highest level of 4D12 binding in the Single Antigen Combi Beads assay. Sequence identity is indicated by dots and amino acids are coloured according to the standard scheme used by RasMol. **(C)** Representative staining (of at least five independent replicates) with W6/32 (top row), or 4D12 (bottom row), of β2-microglobulin-deficient 293T cells transfected with plasmids expressing single chain dimers (SCD) HLA-E*01:03 (first column), HLA-A*11:01 (second column), HLA-B*40:06 (third column), or HLA-C*18:02 (final column). Grey histograms show staining of mock transfected cells.

Although we have previously shown that 3D12 does not cross-react with a panel of 97 representative MHC class I alleles (31 HLA-A, 50 HLA-B, and 16 HLA-C) using the One Lambda LABScreen Single Antigen Class I Combi beads (21), it has been reported that 3D12 can cross-react with HLA-C*17:01 and, to a lesser extent, HLA-C*04:03, HLA-B*27:06 and HLA-B*40:06 (27). Such cross-reactivity seems unlikely as the amino acids at the critical positions 219, 223 and 224 in these alleles differ from those found in HLA-E (**Figure 7A**). We repeated the testing of 3D12 with the Single Antigen Combi beads (**Figure 7B**) and, although the results differed slightly from what we observed previously (most likely due to batch differences in the beads used), only very low levels of binding were again observed. Of the alleles reported to cross-react with 3D12, binding was slightly higher to HLA-B*40:06 than to HLA-C*17:01 (HLA-B*40:06 and HLA-C*17:01; HLA-B*27:06 and HLA-C*04:03 are not included in the panel). Given the artificial nature of this assay, we also tested cross-reactivity of 3D12 for HLA-B*40:06 and HLA-C*17:01 by staining β2-microglobulin-deficient 293T cells expressing single chain dimers (SCD) of these two alleles and observed no significant binding (**Figure 7C**). Therefore, we conclude that 3D12 is indeed specific for HLA-E.For 4D12, it has been reported that this antibody exhibited no cross-reaction with a panel of 60 cell lines from the International Histocompatibility Working Group (23). To allow direct comparison of the cross-reactivity of these antibodies with classical MHC class I alleles, we also tested 4D12 with the Single Antigen Combi beads assay (**Figure 8A**). As with 3D12, most of alleles tested also showed only similar levels of weak binding. However, higher levels of binding were observed with six alleles: HLA-A*11:01, HLA-B*40:06, HLA-C*05:01, HLA-C*08:01, HLA-C*14:02, and HLA-C*18:02. Specific binding of 4D12 to these classical MHC I alleles also seemed unlikely given their sequences differ markedly from that of HLA-E in the 4D12 epitope region (**Figure 8B**). We observed no staining of β2-microglobulin-deficient 293T cells expressing SCD of HLA-A*11:01, HLA-B*40:06 and HLA-C*18:02 (the most cross-reactive HLA-A, B and C alleles – **Figure 8C**), confirming that 4D12 does not cross-react with any of the classical MHC class I alleles tested here.

### 3D12 and 4D12 recognise different forms of HLA-E on the cell surface

**Figure 9:**
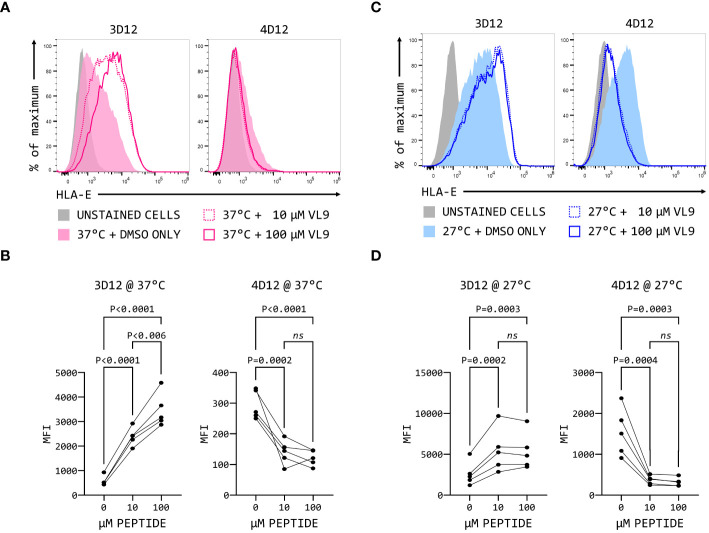
4D12 recognises peptide-free HLA-E. K562 cells over-expressing HLA-E*01:03 were cultured at either 37°C **(A)** or 25°C **(C)**. Cells were pulsed for 4 hours with VL9 peptide at a final concentration of 10 μM (dashed lines) or 100 μM (solid lines), or DMSO (filled histograms). Cells were stained with 3D12 (left histograms) or 4D12 (right histograms). Unstained cells are shown as the solid grey histograms. Five independent replicates were analysed **(B, D)** using a repeated measures ANOVA with Tukey’s correction for multiple comparisons. P values are indicated; ns, not significant.

The fact that 3D12 and 4D12 recognise different epitopes may result in recognition of different forms of HLA-E on the cell surface. When MHC Ia-negative K562 cells over-expressing HLA-E*01:03 were incubated with two different concentrations of VL9 peptide at 37°C, a dose-dependent increase in staining was observed with 3D12 (**Figures 9A, B**, left-hand panels), consistent with the exogenous peptide stabilising HLA-E at the cell surface. In contrast, staining with 4D12 was decreased by the addition of VL9 (**Figures 9A, B**, right-hand panels). Incubating these cells at 27°C to stabilise empty HLA-E at the cell surface (37) increased staining with both 3D12 and 4D12 in the absence of exogenous peptide (**Figure 9C**). Addition of exogenous VL9 peptide at 27°C resulted in a further increase in staining with 3D12 and a decrease in staining with 4D12 (**Figure 9D**). Pulsing cells with an epitope peptide from M. tuberculosis (Mtb44, RLPAKAPLL; 29) gave identical results (**Supplementary Figure 5**), confirming that the changes in 4D12 binding are not specific to the VL9 peptide.

The increase in 3D12 staining in the presence of these peptides results from the stabilisation of existing HLA-E complexes at the cell surface (38). The reduction in staining seen with 4D12 in the presence of exogenous VL9 peptide is most readily explained by this antibody preferentially binding to peptide-free HLA-E complexes that may be present at the cell surface, and which would be converted to correctly folded complexes in the presence of exogenous VL9 peptide. Although peptide-free HLA is typically unstable at the cell surface, we have previously observed that HLA-E can refold *in vitro* more readily in the absence of peptide than classical HLA alleles (29). If empty HLA-E is also more stable than classical MHC class I proteins at the cell surface, it would explain the presence of 4D12-reactive HLA-E at the cell surface.

## Discussion

By employing hybrids of different MHC-E alleles, we have mapped the locations of the epitopes bound by both 3D12, the most commonly used commercially available HLA-E antibody, and 4D12, which binds both HLA-E and certain non-human primate MHC-E alleles. In addition to confirming the previous observation that these antibodies recognise distinct epitopes (23), we also provide evidence to suggest that 4D12 recognises peptide-free HLA-E.

The epitope of 3D12 encompasses a region on the alpha 3 domain containing 4 amino acids that differ between HLA-E and Mamu-E, positions 215, 219, 223 and 224, although only the polymorphisms at positions 219, 223 and 224 appear to be important for either direct interaction with the antibody or maintaining the correct conformation of the epitope. This location is distinct from the two sequences identified by a previous attempt to map the epitope of this antibody (115-QFAYDGKDY-123 and 137-DTAAQI-142; 27). Both of the previously identified sequences are conserved in Mamu-E*02:04, to which 3D12 does not bind, as well as in many HLA-B and C alleles. We note that these authors previously suggested the same two regions for the epitope of the MEM-E/02 antibody (22), a conclusion contradicted by a later study that localised the epitope of this antibody to 54-QEGSEYWDRET-64 (28). The 3D12 epitope defined here corresponds to a region on the alpha 3 domain that overlaps with the binding site of CD8 (positions 223–229; 39). This region is well conserved between classical and non-classical MHC I alleles (**Supplementary Figure 6A**), with specificity for HLA-E the result of the three polymorphic locations identified in our mapping. Glycine at position 223 and histidine at position 224 are unique to HLA-E, with all known Mamu-E and Mafa-E alleles having the aspartic acid and glutamate residues that are present in all known HLA-A, B, C, F and G alleles (**Supplementary Figures 6B, C**). Position 219 also differs between HLA-E and classical MHC class I allele, with the majority of HLA-A, B, C, F and G alleles having arginine rather than the glutamine of HLA-E (the only exceptions being certain HLA-C and G alleles that have tryptophan). Arginine at position 219 also predominates in Mamu-E and Mafa-E, being present in 20 Mamu-E alleles and 12 Mafa-E alleles (of the 22 and 15 alleles, respectively, that have been sequenced across this region). The exceptions are Mamu-E*02:21, Mafa-E*02:08, Mafa-E*02:09 and Mafa-E*02:12, which all have glutamine, and Mamu-E*02:22 which has tryptophan. Further work is required to determine the full extent of the 3D12 contact site, as well which residue contact the antibody, and which are required to maintain the epitope in the correct conformation to allow it to be recognised.

The epitope of 4D12 lies in the alpha 2-1 helix, adjacent to the C terminus of the presented peptide, a region that differs substantially in classical HLA alleles compared with MHC-E (**Supplementary Figure 7A**). In contrast to the 3D12 epitope, the linear nature of the 4D12 epitope allowed a more detailed understanding of the contributions of positions 142, 144 and 151 to the binding of the 4D12. While all three positions are important for binding, the glutamic acid at position 144, which is unique to MHC-E, is the most critical. As with the 3D12 epitope, further mutagenesis will be required to evaluate the contributions to 4D12 binding of the amino acids that neighbour these positions to fully define the exact extent of the epitope.

Although 4D12 has previously been used to stain cells expressing Mamu-E and Mafa-E (13, 24), 4D12 does not bind all Mamu-E alleles. In addition to Mamu-E*02:16, at least four other alleles would be predicted not to be bound by 4D12: Mamu-E*02:08. which has 142T, 144A and 151G, and three alleles (Mamu-E*02:12:01, 02:12:01, and 02:28) which have144A and 151G (**Supplementary Figure 7B**). For Mafa-E (**Supplementary Figure 7C**), while all 16 known alleles have isoleucine at position 142 and glutamate at position 144, one allele (Mafa-E*02:15:01:01) has S151G, although this difference would not be expected to abolish 4D12 binding. Finally, two Mamu-E alleles (Mamu-E*02:05 and Mamu-E*0221) and three Mafa-E alleles (Mafa-E*02:08, 02:09 and 02:12) have arginine rather than glutamate at position 145, and the effects of this difference on 4D12 binding remain to be determined.

The reduced 4D12 staining of the K562E cells in the presence of the VL9 peptide (**Figure 9**) suggests that this antibody may preferentially bind to peptide-free forms of HLA-E, something that has been suggested previously (40). Although such discrimination is unexpected given 4D12 recognises a linear epitope, it may result from a steric clash between the antibody and the peptide. Alternatively, occupancy of the peptide binding groove may alter the conformation of the alpha 2 domain compared with empty or denatured HLA-E, remodelling it in a way that reduces or precludes antibody binding. Such behaviour would parallel that of the antibody 64-3-7, which binds to positions 46–52 (EPQAPWM) on the alpha 1 of empty forms of the mouse H-2L^d^ molecule (41, 42). Structural analysis of the 64-3-7 bound to a peptide containing its epitope (43, 44) has shown amino acids in the core of the epitope (Q48, P50, and W51) contact the antibody. When peptide is bound by H2-L^d^, the conformation of the epitope changes, and the side chains of these amino acids become inaccessible.

4D12 binding to peptide-free MHC-E may appear to be at odds with the fact that it readily stains unpermeabilised cells expressing HLA-E, as empty HLA is thought to be unstable at the cell surface (37). However, we have previously shown that the stability of the VL9 peptide in HLA-E is relatively modest compared with that of peptides presented by classical HLA molecules (45). Moreover, HLA-E appears to be more stable in the absence of peptide than classical MHC class I molecules (29). Peptide-free HLA-E may be present at the cell surface in relatively high amounts compared with peptide-free classical MHC class I molecules, explaining why 4D12 surface-stains cells expressing MHC-E. Interaction of the CD94/NKG2 family of receptors with HLA-E is absolutely dependent on the presence of the VL9 peptide (8), so the presence of empty HLA-E at the cell surface would not interfere with NK cell surveillance. Structural changes in the alpha 2 helix in the region of the 4D12 epitope resulting from peptide binding may also be of relevance to the known tapasin-dependence of HLA-E (5), although further work is required to understand both the nature and significance of these changes.

Weak binding of the VL9 peptide and a possible increase in the stability of peptide-free HLA-E would also be predicted to make the HLA-E SCT more dynamic than SCT of classical MHC class I alleles. The covalently linked peptide may be able to dissociate and re-associate without the protein being lost from the cell surface. 4D12 staining of cells expressing MHC-E SCT would then result from the transient presence of these “peptide free” SCT at the cell surface. However, an alternative explanation for the binding of 4D12 to cells expressing MHC-E SCT is suggested by the crystal structure of HLA-E presenting a peptide extended at the C-terminus (to allow formation of a disulphide bond with a cysteine at position 84). In this complex, the alpha 2 domain in the region of the 4D12 epitope shows significant distortion (46). Although the published structures of conventional MHC SCT show the expected conformation of the heavy chain (for example, see 47), there are no published crystal structures of HLA-E SCT. If distortion of the alpha 2 domain is common to all MHC-E complexes when the peptide sequence is extended (such as in SCT), 4D12 may stain cells expressing MHC-E SCT because this alpha 2 distortion holds the 4D12 epitope in a conformation that allows the antibody to bind. Significant distortion of the alpha 2 helix will be a particular concern if HLA-E SCT are to be used for functional assays related to antigen presentation, and further structural analysis is warranted to determine the suitability of HLA-E SCT is these assays.

The LABScreen beads assay (**Figure 8A**) suggested that 4D12 cross-reacted at a low level with certain HLA-A, B and C alleles, but staining of cells expressing SCD of the most cross-reactive alleles was negative (**Figure 8C**). Consistent with this lack of cross-reaction, although all the tested alleles have isoleucine at position 142, none have serine at position 151, or the critical glutamate at position 144 (**Supplementary Figure 8**). Moreover, the sequence across the region bound by 4D12 in those alleles that gave the highest 4D12 binding in the LABScreen beads assay is identical in other alleles that show much less cross-reaction (for example, HLA-A*11:01 and HLA-A*11:02, HLA-B*40:06 and HLA-B*40:02, and HLA-C*18:02 and HLA-C*01:02). It is known that the beads used are coated with a mix of correctly folded and mis-folded protein (48), while peptide-free forms of classical HLAs would not be expected to be present at the cell surface. Therefore, the level of cross-reaction in the LABScreen beads assay likely reflects the amounts of mis-folded protein present on the bead. Consistent with this, the same 6 alleles that gave the highest levels of cross-reaction with 4D12 also gave the highest levels of cross-reaction with 3D12, albeit at lower levels.

Both 3D12 and 4D12 bind HLA-E on the opposite side to β2-microglobulin, recognising epitopes formed from only the HLA-E heavy chain. This contrasts with the pan-HLA antibody W6/32, which has a discontinuous epitope involving both the HLA heavy chain and β2-microglobulin (35). Therefore, the binding of W6/32 to classical class I proteins at the cell surface is likely to be limited to correctly folded complexes, as these are known to rapidly dissociate upon loss of the presented peptide (37). In contrast, our results suggest that neither 4D12 nor 3D12 are suited to measuring surface expression levels of trimeric MHC-E/β2m/peptide complexes. This is especially true for 4D12, where staining levels will actually give a measure of the amount of peptide-free MHC-E at the cell surface. Given 4D12 is the only commercially available antibody available that recognises at least some non-human primate MHC-E alleles, this is a particular concern for non-human primate studies.

With 3D12, the conformational nature of the epitope may impose an element of selectivity in terms of the forms of HLA-E to which the antibody can bind, and the increased staining seen when cells are incubated with the VL9 peptide is consistent with the recognition of HLA-E presenting peptide. The increased 3D12 staining seen when cells are incubated at 27°C may suggests that this antibody may also recognise peptide-free forms of HLA-E, and that 3D12 staining may give a measure of the total expression of all forms of HLA-E present at the cell surface. However, it is likely that lower temperatures will also stabilise trimeric HLA-E/β2-microglobulin/peptide complexes at the cell surface, so the binding of 3D12 to peptide-free HLA-E remains be confirmed. The proximity of the 3D12 epitope to the plasma membrane may also mean that binding of 3D12 could be affected by changes in the lipid composition of the membrane, as observed previously for the pan-MHC class I antibody W6/32 (25). Therefore, further definition of the exact range of complexes that 3D12 can recognise, and how its staining may be affected by membrane composition, will be essential to fully determine the utility of this antibody for monitoring cell surface expression of correctly folded HLA-E/β2-microglobulin/peptide complexes.

Despite these concerns about the exact forms of HLA-E recognised by 3D12, we have previously used it as the capture antibody in a sandwich ELISA to measure peptide binding by HLA-E (49). In this assay, the amount of HLA-E/β2-microgobulin/peptide complex generated in a micro-scale refold, captured by 3D12, and detected using an antibody against β2-microglobulin, depends on the affinity of HLA-E for the peptide. Attempts to set up the same ELISA for Mamu-E using 4D12 have been unsuccessful (G. Gillespie, L. Walters and A.J.M., unpublished data), most likely because this antibody only recognises peptide-free complexes. To overcome the lack of a suitable Mamu-E-specific capture antibody, we plan to trial a peptide binding ELISA using Mamu-E*02:04 mutated at positions 219, 223 and 224 to allow it to be bound by 3D12. The location of these mutations (on the alpha 3 domain) would not be expected to have a significant impact on peptide binding, and we feel that this represents a better alternative to the approach adopted by a recently published Mamu-E peptide binding ELISA (50), which used streptavidin to capture biotinylated Mamu-E/β2-microglobulin/peptide complexes. Should this approach prove successful, it will be informative to extend it to other alleles of Mamu-E to determine if the polymorphic nature of this MHC has any impact on its peptide repertoire.

In conclusion, we have determined the locations on HLA-E of the epitopes bound by the antibodies 3D12 and 4D12, and present evidence that 4D12 is specific for peptide-free HLA-E. Our results also suggest that 3D12 may also recognise more than just canonical HLA-E/β2m/peptide complexes, although further experiments are necessary to elucidate the exact forms of HLA-E that are recognised by this antibody.

## Data availability statement

The original contributions presented in the study are included in the article/**Supplementary Material**. Further inquiries can be directed to the corresponding author.

## Ethics statement

Ethical approval was not required for the studies on humans in accordance with the local legislation and institutional requirements because only commercially available established cell lines were used.

## Author contributions

SB: Conceptualization, Data curation, Formal analysis, Investigation, Methodology, Visualization, Writing – original draft, Writing – review & editing. NJ: Data curation, Formal analysis, Investigation, Writing – review & editing. KF: Conceptualization, Formal analysis, Funding acquisition, Methodology, Resources, Supervision, Writing – review & editing. PB: Conceptualization, Funding acquisition, Methodology, Resources, Supervision, Writing – review & editing. AM: Conceptualization, Funding acquisition, Resources, Supervision, Writing – review & editing.
