# Supplementary material for: The antibodies 3D12 and 4D12 recognise distinct epitopes and conformations of HLA-E
Source: Front Immunol. 2024 Mar 20;15:1329032. doi: 10.3389/fimmu.2024.1329032 (PMC10987726; doi:10.3389/fimmu.2024.1329032)
Supplement: Supplementary file 1 [file DataSheet_1.pdf]

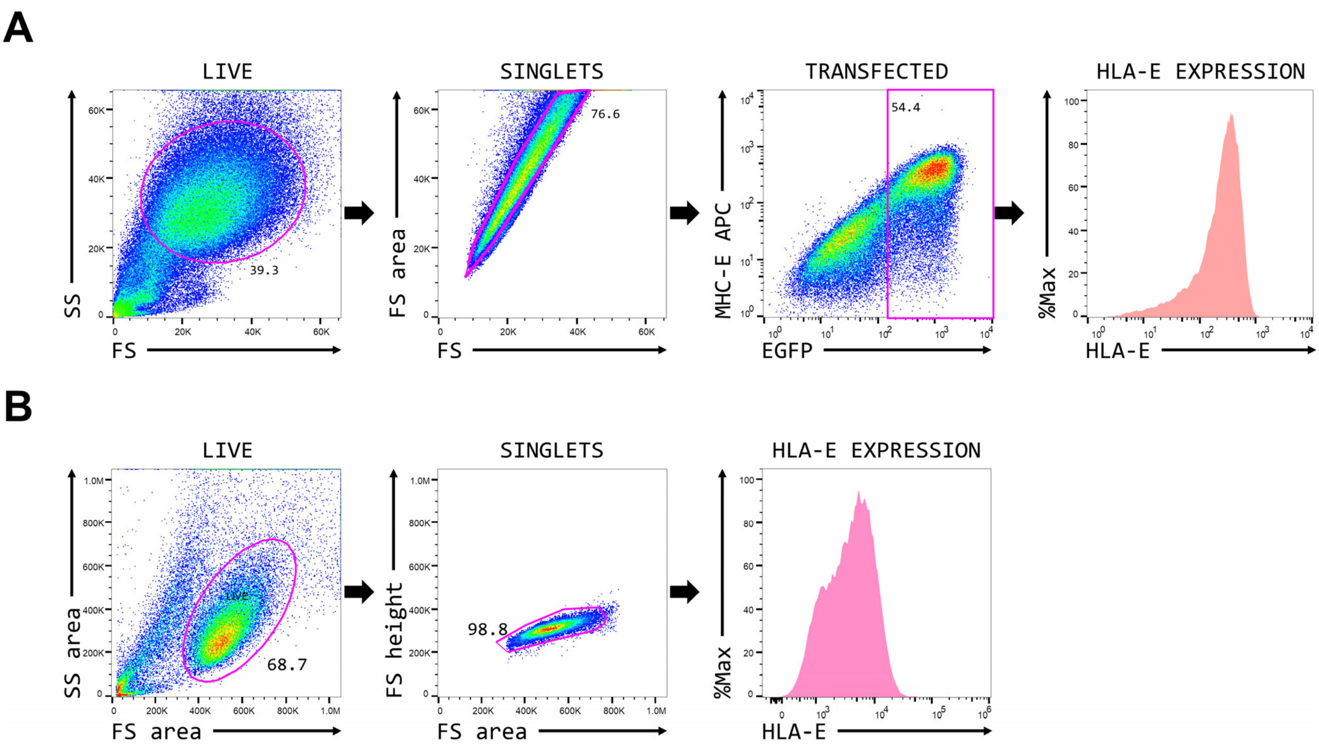

SUPPLEMENTARY FIGURE 1:

(A) Gating strategy used on the CyAn ADP for single chain trimer (SCT) transfected β2-microglobulin-deficient 293T cells: cells were gated, and debris excluded, on the basis of Side Scatter (SS) versus Forward Scatter (FS), single cells defined by FS (area) versus FS (linear), and transfected cells identified by EGFP expression. The example shown is gating of cells transfected with the HLA-E*01:03 VL9 SCT. **(B)** Gating strategy used on the Attune NXT for the K562 cells over-expressing HLA-E*01:03: cells were gated, and debris excluded, on the basis of SS (area) versus FS (area), and single cells defined by FS (height) versus FS (area).

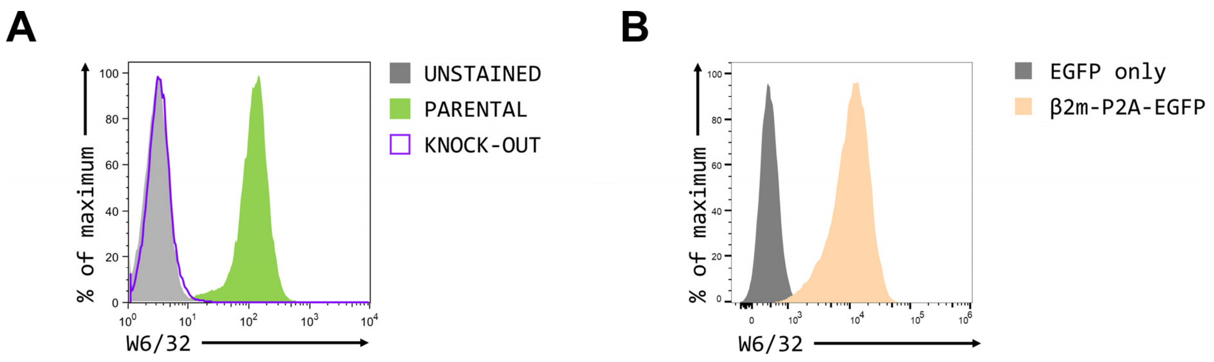

SUPPLEMENTARY FIGURE 2:

(A) Representative W6/32 staining (of three independent replicates) of the parental 293T cells (filled green histogram) and the β2-microglobulin-deficient 293T cells (purple line). Unstained cells are shown as the filled grey histogram. **(B)** MHC class I expression can be restored in β2-microglobulin-deficient 293T cells by transient expression of a plasmid expressing β2-microglobulin. The histograms (representative of three independent replicates) show W6/32 staining of EGFP positive cells transfected with constructs expressing either only EGFP (dark grey histogram), or β2-microglobulin and EGFP linked by the picornavirus 2A slip sequence (orange histogram).

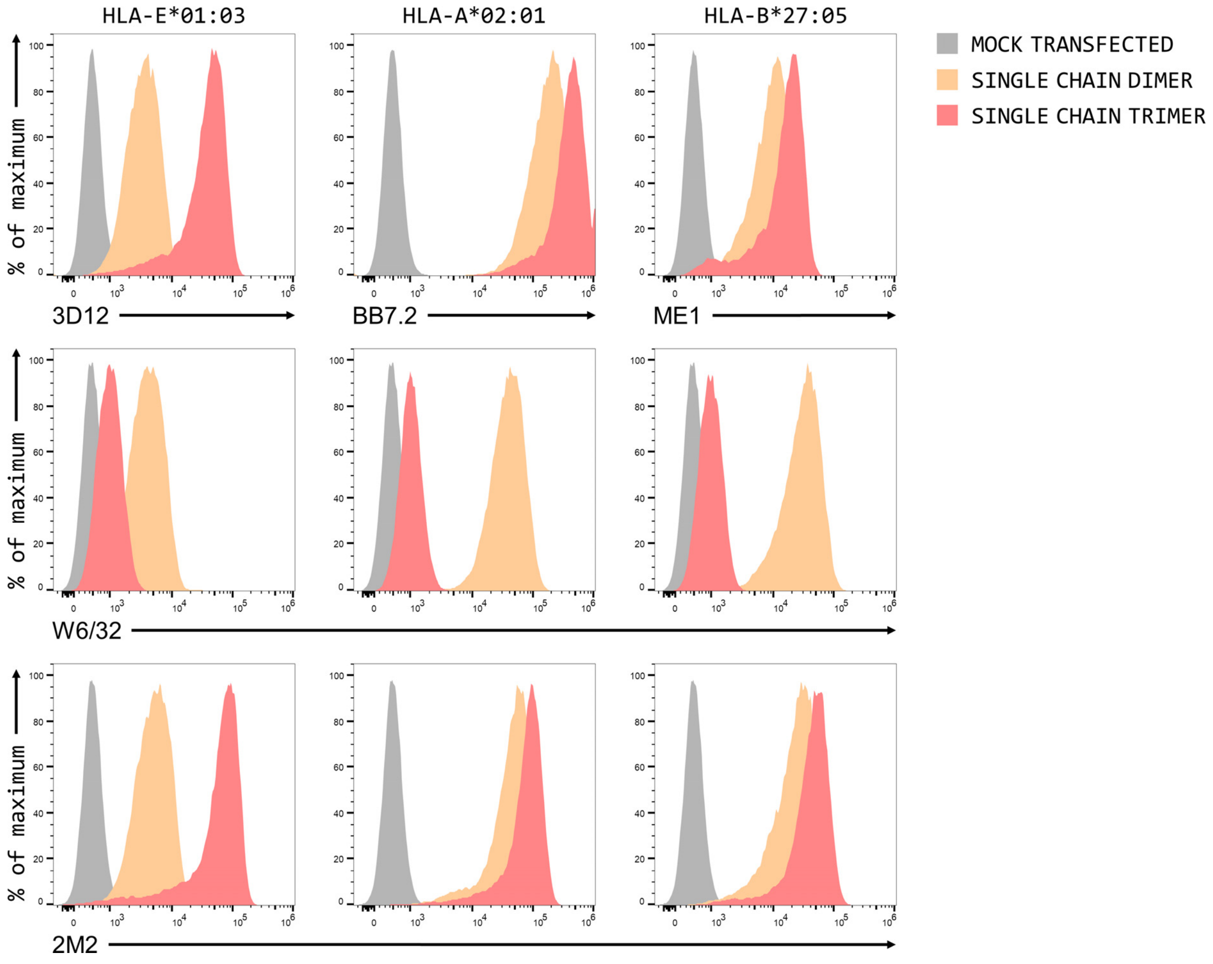

SUPPLEMENTARY FIGURE 3:

Comparison of staining of the β2-microglobulin-deficient 293T cells expressing single chain dimers (SCD; filled orange histograms) and single chain trimers (SCT; filled red histograms) of HLA-E*01:03 (left column), HLA-A*02:01 (middle column) and HLA-B*27:05 (right column). TOP ROW: allele-specific antibodies (3D12 for HLA-E*01:03, BB7.2 for HLA-A*02:01, and ME1 for HLA-B*27:05). MIDDLE ROW: the pan-HLA antibody W6/32. BOTTOM ROW: the β2-microglobulin-specific antibody 2M2. Staining of the cells expressing the SCT is higher than that of the cells expressing the SCD with 2M2 and the three allele-specific antibodies. For W6/3, staining of the cells expressing the SCT is always lower than that of cells expressing the SCD. Histograms shown are representative of at least three independent replicates.

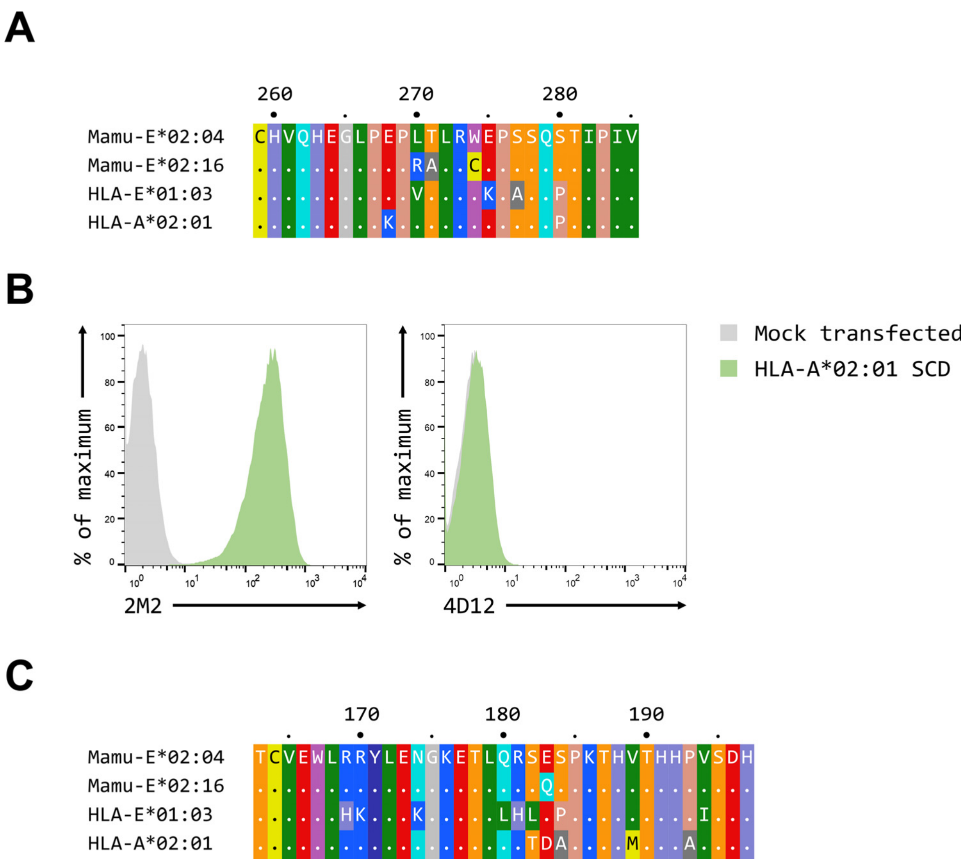

SUPPLEMENTARY FIGURE 4:

(A) Comparison of the sequences of Mamu-E*02:04, Mamu-E*02:16, HLA-E*01:03, and HLA-A*02:01 around the position 270, 271 and 274 polymorphisms between the two Mamu-E alleles. Sequence identity with Mamu-E*02:04 is shown by dots and amino acids are coloured according to the standard scheme used by RasMol. The lack of sequence conservation with HLA-E in this region, coupled with the similarity of the sequences of Mamu-E*02:04 and HLA-A*02:01, suggested that this was not the location of the 4D12 epitope. **(B)** 4D12 does not stain cells expressing HLA-A*02:01. Representative staining (of three independent replicates) with a β2-microglobulin-specific antibody (2M2, left hand panel), or 4D12 (right hand panel), of β2-microglobulin-deficient 293T cells transfected with plasmids expressing either an irrelevant protein (grey histograms) or a single chain dimer (SCD) of HLA-A*02:01 and β2-microglobulin (green histograms). **(C)** Comparison of the sequences of Mamu-E*02:04, Mamu-E*02:16, HLA-E*01:03, and HLA-A*02:01 around the position 183 polymorphism between the two Mamu-E alleles. Sequence identity with Mamu-E*02:04 is shown by dots and amino acids are coloured according to the standard scheme used by RasMol. The lack of sequence conservation with HLA-E in this region again suggested that this was not the location of the 4D12 epitope.

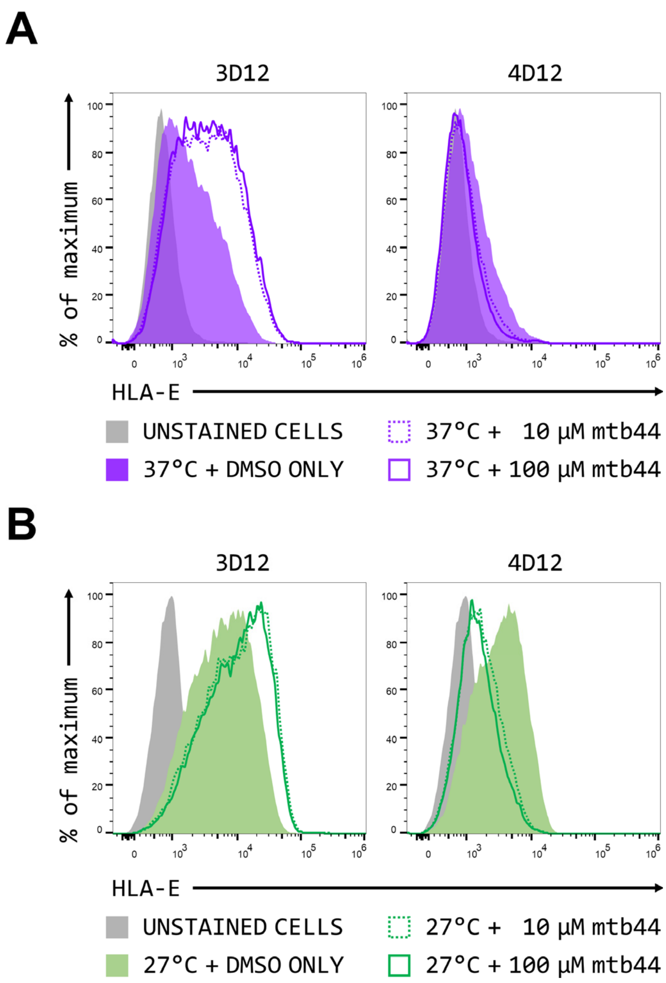

SUPPLEMENTARY FIGURE 5:

K562 cells over-expressing HLA-E*01:03 were cultured at either 37°C (panel **A**) or 27°C (panel **C**). Cells were pulsed for 4 hours with mtb44 peptide (RLPAKAPLL) at a final concentration of 10 μM (dashed lines) or 100 μM (solid lines), or DMSO (filled histograms). Cells were stained with 3D12 (left histograms) or 4D12 (right histograms). Unstained cells are shown as the solid grey histograms, and data is representative of three independent replicates.

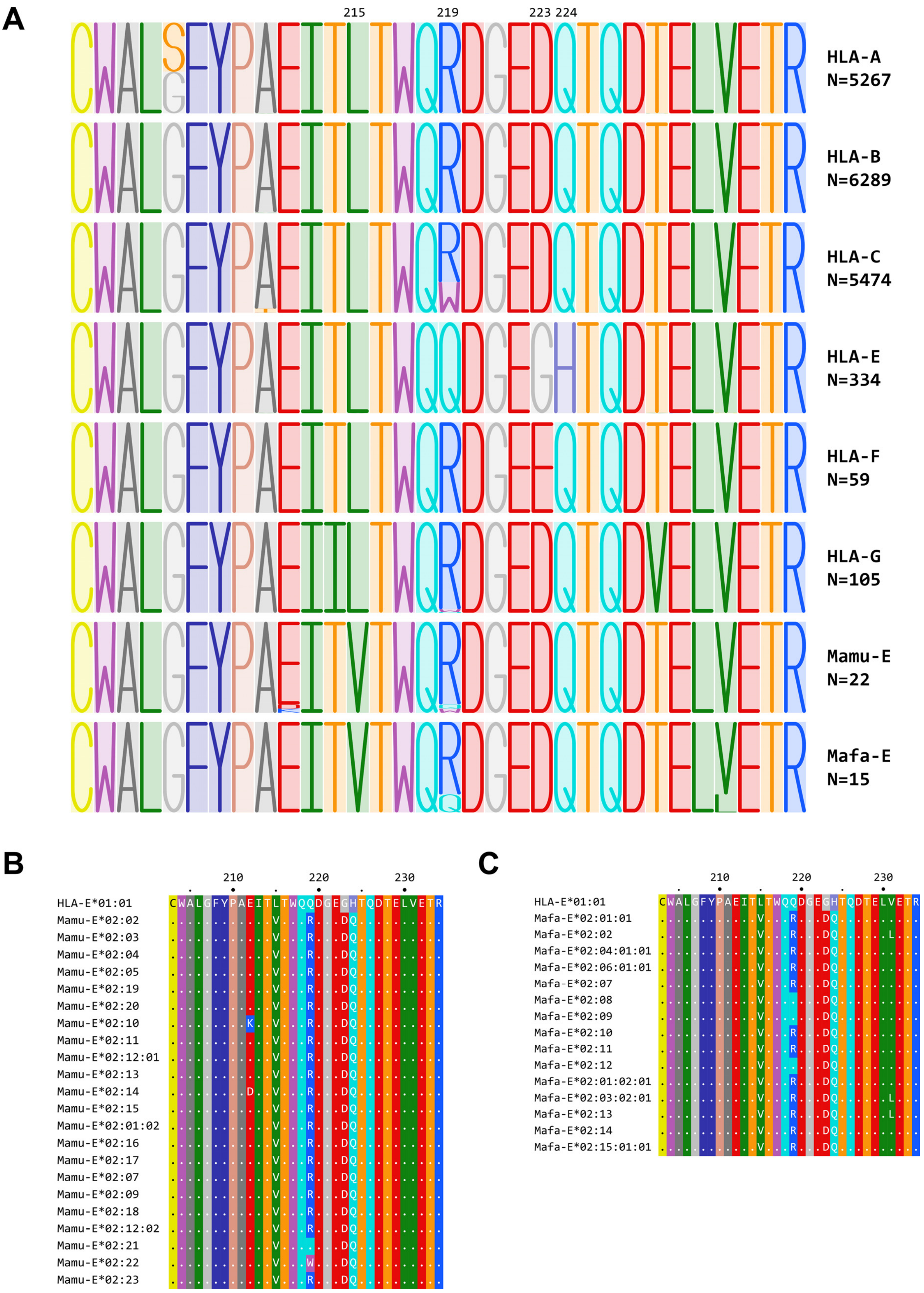

SUPPLEMENTARY FIGURE 6:

(A) Sequence logos of the region (positions 203 to 234) containing the 3D12 epitope for all sequenced alleles of HLA-A, B, C, E, F, and G in the IMGT HLA Database (release 3-52), and all sequenced Mamu-E and Mafa-E alleles in the IMGT MHC Database (release 3-11-0-0). The number of sequences for each locus is indicated. **(B, C)** Comparison of the sequences (positions 203 to 234) of HLA-E*01:03 and all sequenced Mamu-E and Mafa-E alleles from the IMGT MHC Database (release 3-11-0-0). Sequence identity with HLA-E*01:03 is shown by dots and amino acids are coloured according to the standard scheme used by RasMol.

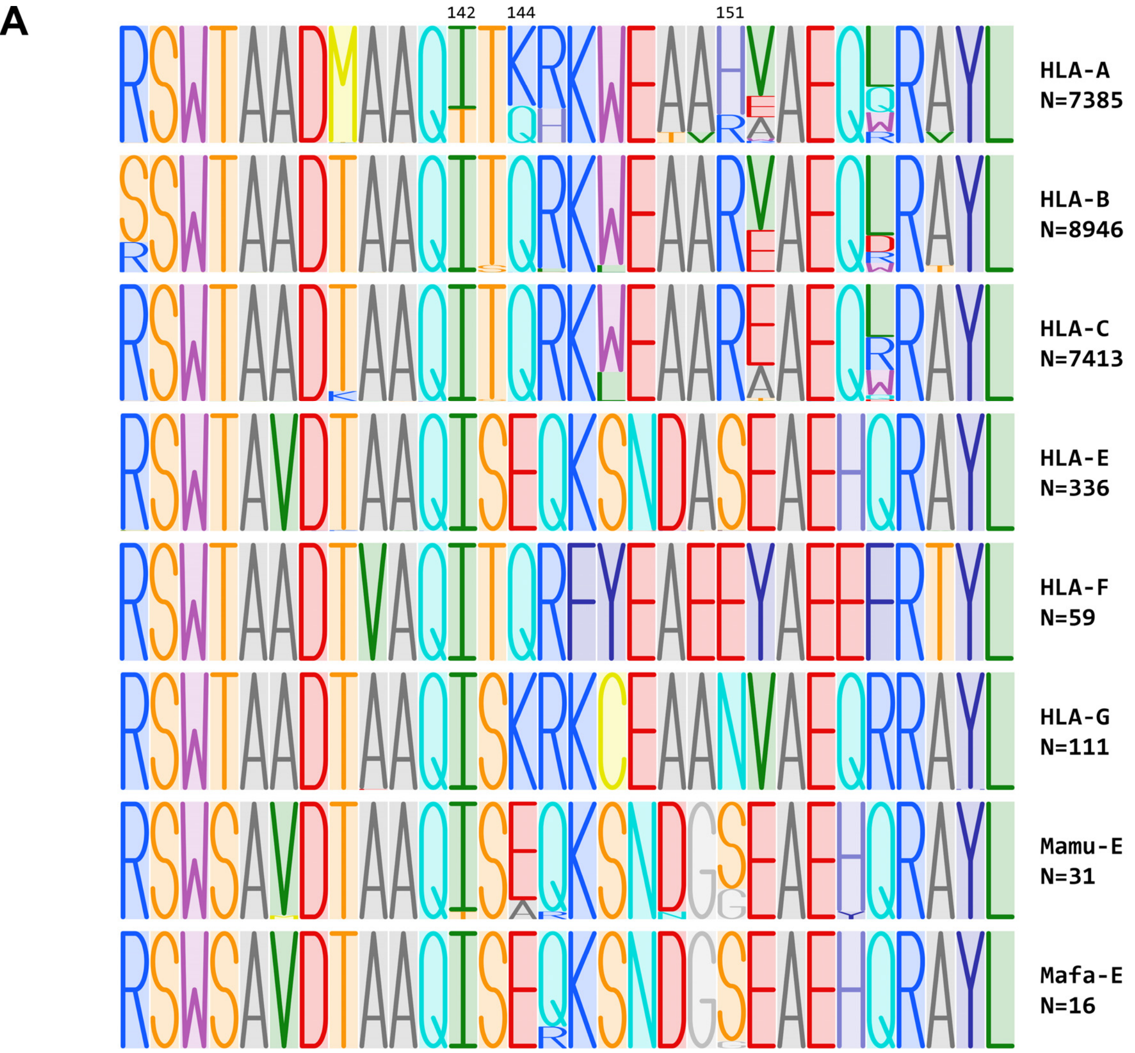

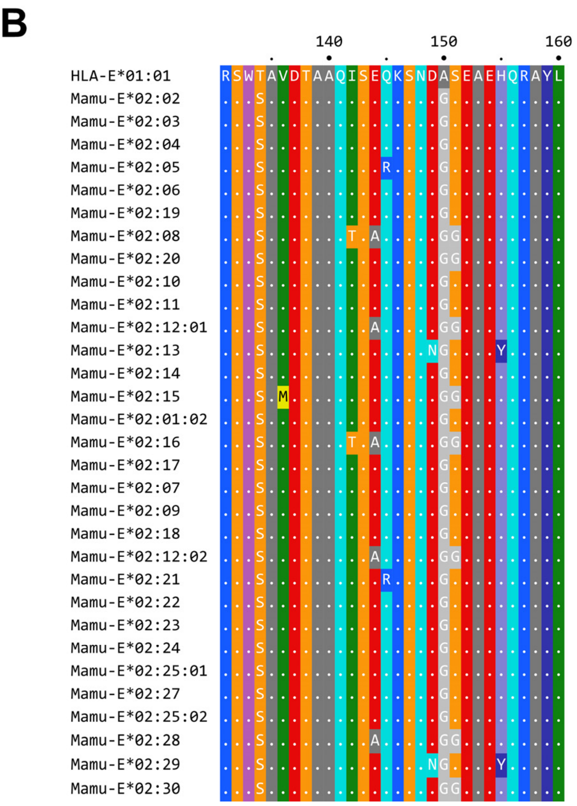

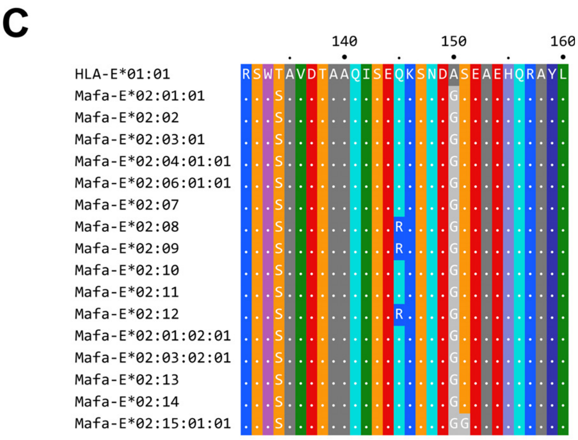

SUPPLEMENTARY FIGURE 7:

(A) Sequence logos of the region (positions 131 to 160) containing the 4D12 epitope for all sequenced alleles of HLA-A, B, C, E, F, and G in the IMGT HLA Database (release 3-52), and all sequenced Mamu-E and Mafa-E alleles in the IMGT MHC Database (release 3-11-0-0). The number of sequences for each locus is indicated. **(B, C)** Comparison of the sequences (positions 131 to 160) of HLA-E*01:03 and all sequenced Mamu-E and Mafa-E alleles from the IMGT MHC Database (release 3-11-0-0). Sequence identity with HLA-E*01:03 is shown by dots and amino acids are coloured according to the standard scheme used by RasMol.

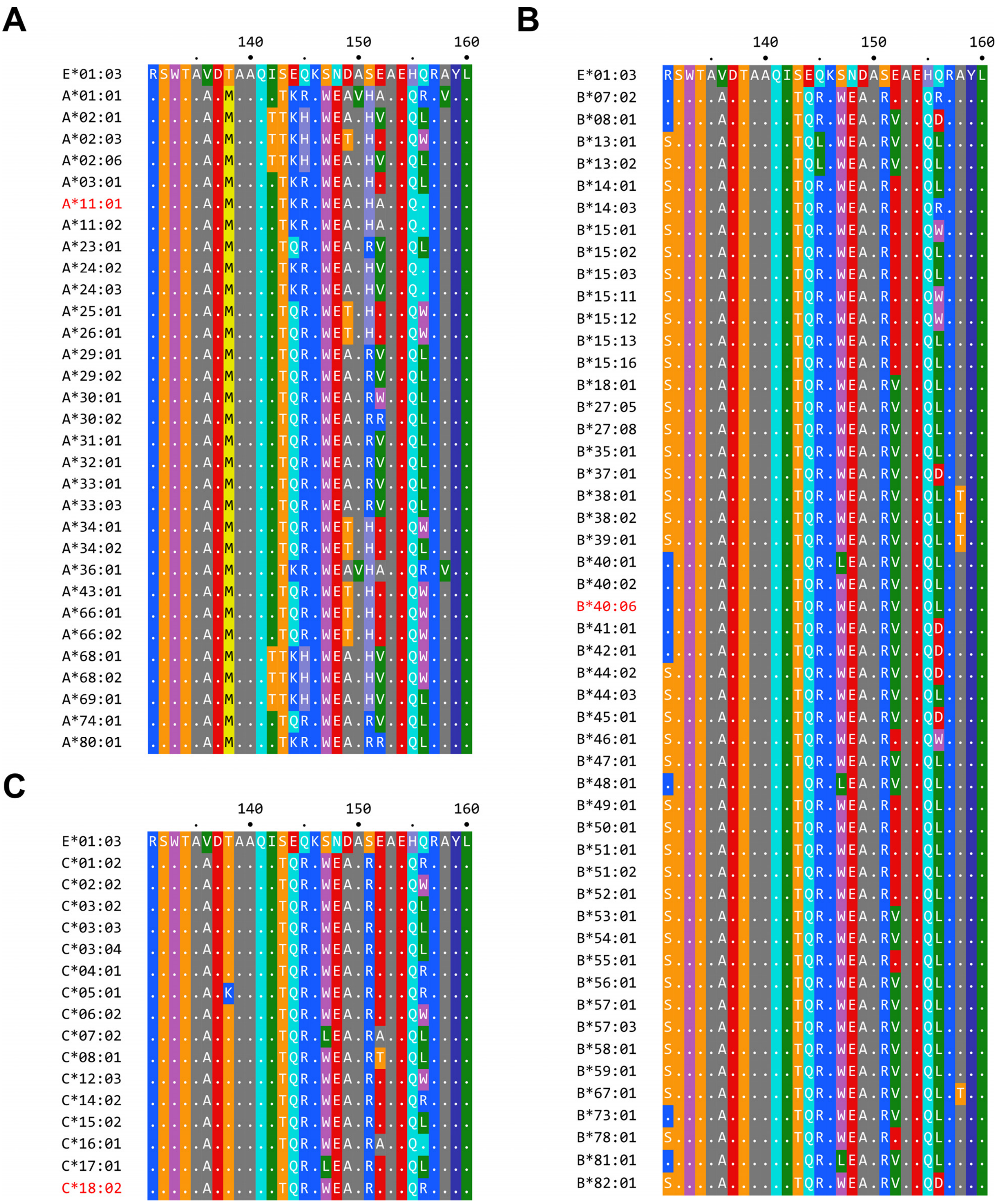

SUPPLEMENTARY FIGURE 8:

Sequence alignments of the region (positions 131 to 160) containing the 4D12 epitope for the 31 HLA-A (panel **A**), 50 HLA-B (panel **B**), and 16 HLA-C (panel **C**) alleles tested for cross-reaction using the LABscreen Single Antigen Combi bead assay. Sequence identity with HLA-E*01:03 is shown by dots, and amino acids are coloured according to the standard scheme used by RasMol. The HLA-A, HLA-B and HLA-C alleles (HLA-A*11:01, HLA-B*4006, and HLA-C*18:02) with the highest level of apparent cross-reaction are indicated in red.
