# Supplementary material for: The antibodies 3D12 and 4D12 recognise distinct epitopes and conformations of HLA-E
Source: Front Immunol. 2024 Mar 20;15:1329032. doi: 10.3389/fimmu.2024.1329032 (PMC10987726; doi:10.3389/fimmu.2024.1329032)
Supplement: Supplementary file 2 [file Table_1.docx]

## SUPPLEMENTARY TABLE 1: MHC-E extracellular domain primers

| PRIMER NAME | SEQUENCE (5'–3') | PARTNER PRIMER |
| --- | --- | --- |
| MHC-E ECD F | GACCTGGGCGGGCTCCCACTCCTTGAAGTATTTCC | HLA-E/Mamu-E ECD R or HLA-E/Mamu-E mutagenic R primer |
| HLA-E ECD R | GTGGATCCAAGCTGTGAGACTCAGACCC | HLA-E ECD F or HLA-E mutagenic F primer |
| MAMU-E*02:04 ECD R | GTGGATCCGGTTTATAAGCTGTGAGAGAATCATCAGATCCC | MHC-E ECD F or Mamu-E mutagenic F primer |
| MAMU-E*02:16 ECD R | GTGGATCCGTTTTATAAGCTGTGAGAGACTCATCAGATCCC | MHC-E ECD F or Mamu-E mutagenic F primer |

**NOTE:**

Mutations were introduced by amplifying an appropriate template with MHC-E ECD F and a mutagenic reverse primer, or a mutagenic F primer and HLA-E/Mamu-E ECD R. Amplification products were purified, mixed, reamplified with MHC-E ECD F and HLA-E/Mamu-E ECD R, and the resulting mutated sequence was cloned into the single chain trimer expression plasmids using PpuMI and SbfI.
