# Supplementary material for: The antibodies 3D12 and 4D12 recognise distinct epitopes and conformations of HLA-E
Source: Front Immunol. 2024 Mar 20;15:1329032. doi: 10.3389/fimmu.2024.1329032 (PMC10987726; doi:10.3389/fimmu.2024.1329032)
Supplement: Supplementary file 3 [file Table_2.docx]

## SUPPLEMENTARY TABLE 2: HLA-E mutagenic primers

| mutation | PRIMER | PRIMER SEQUENCE (5'–3') | PARTNER PRIMER | TEMPLATE |
| --- | --- | --- | --- | --- |
| I142T | Fwd | CACGGCGGCTCAGACTTCCGAGCAAAAGTCAAATGATG | HLA-E ECD R | HLA-E WT |
|  | Rev | CATCATTTGACTTTTGCTCGGAAGTCTGAGCCGCCGTG | MHC-E ECD F |  |
| E144A | Fwd | CACGGCGGCTCAGATCTCCGCTCAAAAGTCAAATGATG | HLA-E ECD R | HLA-E WT |
|  | Rev | CATCATTTGACTTTTGAGCGGAGATCTGAGCCGCCGTG | MHC-E ECD F |  |
| S151G | Fwd | GTCAAATGATGCCGGTGAGGCGGAGCACCAG | HLA-E ECD R | HLA-E WT |
|  | Rev | CTGGTGCTCCGCCTCACCGGCATCATTTGAC | MHC-E ECD F |  |
| I142T+E144A | Fwd | CACGGCGGCTCAGACTTCCGCTCAAAAGTCAAATGATG | HLA-E ECD R | HLA-E WT |
|  | Rev | CATCATTTGACTTTTGAGCGGAAGTCTGAGCCGCCGTG | MHC-E ECD F |  |
| I142T+S151G | Fwd | *As HLA-E S151G Fwd* | HLA-E ECD R | HLA-E I142T |
|  | Rev | *As HLA-E S151G Rev* | MHC-E ECD F |  |
| E144A+S151G | Fwd | *As HLA-E S151G Fwd* | HLA-E ECD R | HLA-E E144A |
|  | Rev | *As HLA-E S151G Rev* | MHC-E ECD F |  |
| I142T+E144A+S151G | Fwd | *As HLA-E S151G Fwd* | HLA-E ECD R | HLA-E I142T+E144A |
|  | Rev | *As HLA-E S151G Rev* | MHC-E ECD F |  |
| L215V | Fwd | CTGCGGAGATCACAGTGACCTGGCAGC | HLA-E ECD R | HLA-E WT |
|  | Rev | GCTGCCAGGTCACTGTGATCTCCGCAG | MHC-E ECD F |  |
| Q219R | Fwd | GACCTGGCAGCGGGATGGGGAGG | HLA-E ECD R | HLA-E WT |
|  | Rev | CCTCCCCATCCCGCTGCCAGGTC | MHC-E ECD F |  |
| G223D | Fwd | GGATGGGGAGGACCATACCCAGGACAC | HLA-E ECD R | HLA-E WT |
|  | Rev | GTGTCCTGGGTATGGTCCTCCCCATC | MHC-E ECD F |  |
| H224Q | Fwd | GGATGGGGAGGGCCAGACCCAGGACAC | HLA-E ECD R | HLA-E WT |
|  | Rev | GTGTCCTGGGTCTGGCCCTCCCCATCC | MHC-E ECD F |  |
| L215V+Q219R | Fwd | *As HLA-E Q219R Fwd* | HLA-E ECD R | HLA-E L215V |
|  | Rev | *As HLA-E Q219R Rev* | MHC-E ECD F |  |
| G223D+H224Q | Fwd | GGATGGGGAGGACCAGACCCAGGACAC | HLA-E ECD R | HLA-E WT |
|  | Rev | GTGTCCTGGGTCTGGTCCTCCCCATCC | MHC-E ECD F |  |

**NOTE:** Mutated codons are underlined.
