# Supplementary material for: The antibodies 3D12 and 4D12 recognise distinct epitopes and conformations of HLA-E
Source: Front Immunol. 2024 Mar 20;15:1329032. doi: 10.3389/fimmu.2024.1329032 (PMC10987726; doi:10.3389/fimmu.2024.1329032)
Supplement: Supplementary file 4 [file Table_3.docx]

## SUPPLEMENTARY TABLE 3: Mamu-E*02:16 mutation primers

| MUTATION | PRIMER | PRIMER SEQUENCE (5'–3') | PARTNER PRIMER | TEMPLATE |
| --- | --- | --- | --- | --- |
| T142I | Fwd | *As HLA-E E144A Fwd* | Mamu-E ECD R | Mamu-E*02:16 WT |
|  | Rev | *As HLA-E E144A Rev* | MHC-E ECD F |  |
| A144E | Fwd | *As HLA-E I142T Fwd* | MAMU-E ECD R | Mamu-E*02:16 WT |
|  | Rev | *As HLA-E I142T Rev* | MHC-E ECD F |  |
| G151S | Fwd | GTCAAATGATGGCTCTGAGGCGGAGCACCAG | MAMU-E ECD R | Mamu-E*02:16 WT |
|  | Rev | CTGGTGCTCCGCCTCAGAGCCATCATTTGAC | MHC-E ECD F |  |
| T142I+A144E | Fwd | CACGGCGGCTCAGATCTCCGAGCAAAAGTCAAATGATG | MAMU-E ECD R | Mamu-E*02:16 T142I+A144E |
|  | Rev | CATCATTTGACTTTTGCTCGGAGATCTGAGCCGCCGTG | MHC-E ECD F |  |
| T142I+G151S | Fwd | *As Mamu-E*02:16 G151S Fwd* | MAMU-E ECD R | Mamu-E*02:16 T142I |
|  | Rev | *As Mamu-E*02:16 G151S Rev* | MHC-E ECD F |  |
| A144E+G151S | Fwd | *As Mamu-E*02:16 G151S Fwd* | MAMU-E ECD R | Mamu-E*02:16 A144E |
|  | Rev | *As Mamu-E*02:16 G151S Rev* | MHC-E ECD F |  |
| T142I+A144E+G151S | Fwd | *As Mamu-E*02:16 G151S Fwd* | MAMU-E ECD R | Mamu-E*02:16 T142I+A144E |
|  | Rev | *As Mamu-E*02:16 G151S Rev* | MHC-E ECD F |  |

**NOTE:** Mutated codons are underlined.
